# Teriflunomide attenuates neuroinflammation-related neural damage in mice carrying human *PLP1* mutations

**DOI:** 10.1186/s12974-018-1228-z

**Published:** 2018-07-03

**Authors:** Janos Groh, Michaela Hörner, Rudolf Martini

**Affiliations:** Department of Neurology, Section of Developmental Neurobiology, University Hospital Wuerzburg, D-97080 Wuerzburg, Germany

**Keywords:** Proteolipid protein, Axonal degeneration, Inflammation, T-lymphocytes, Teriflunomide

## Abstract

**Background:**

Genetically caused neurological disorders of the central nervous system (CNS) are mostly characterized by poor or even fatal clinical outcome and few or no causative treatments are available. Often, these disorders are associated with low-grade, disease-promoting inflammation, another feature shared by progressive forms of multiple sclerosis (PMS). We previously generated two mouse lines carrying distinct mutations in the oligodendrocytic *PLP1* gene that have initially been identified in patients diagnosed with MS. These mutations cause a loss of PLP function leading to a histopathological and clinical phenotype common to both PMS and genetic CNS disorders, like hereditary spastic paraplegias. Importantly, neuroinflammation promotes disease progression in these models, suggesting that pharmacological modulation of inflammation might ameliorate disease outcome.

**Methods:**

We applied teriflunomide, an approved medication for relapsing-remitting MS targeting activated T-lymphocytes, in the drinking water (10 mg/kg body weight/day). Experimental long-term treatment of PLP mutant mice was non-invasively monitored by longitudinal optical coherence tomography and by rotarod analysis. Immunomodulatory effects were subsequently analyzed by flow cytometry and immunohistochemistry and treatment effects regarding neural damage, and neurodegeneration were assessed by histology and immunohistochemistry.

**Results:**

Preventive treatment with teriflunomide attenuated the increase in number of CD8+ cytotoxic effector T cells and fostered the proliferation of CD8+ CD122+ PD-1+ regulatory T cells in the CNS. This led to an amelioration of axonopathic features and neuron loss in the retinotectal system, also reflected by reduced thinning of the innermost retinal composite layer in longitudinal studies and ameliorated clinical outcome upon preventive long-term treatment. Treatment of immune-incompetent PLP mutants did not provide evidence for a direct, neuroprotective effect of the medication. When treatment was terminated, no rebound of neuroinflammation occurred and histopathological improvement was preserved for at least 75 days without treatment. After disease onset, teriflunomide halted ongoing axonal perturbation and enabled a recovery of dendritic arborization by surviving ganglion cells. However, neither neuron loss nor clinical features were ameliorated, likely due to already advanced neurodegeneration before treatment onset.

**Conclusions:**

We identify teriflunomide as a possible medication not only for PMS but also for inflammation-related genetic diseases of the nervous system for which causal treatment options are presently lacking.

**Electronic supplementary material:**

The online version of this article (10.1186/s12974-018-1228-z) contains supplementary material, which is available to authorized users.

## Background

Genetic defects primarily affecting myelinating glia cells of the central nervous system (CNS) often cause not only glial but also axonal damage [1–4]. While disturbed axon-glia metabolic coupling is likely involved in glia-mediated axonal perturbation [5], inflammatory mechanisms induced by cell-autonomic consequences of glial mutations are other important amplifiers of axonal damage in myelin mutants [4, 6, 7].

We have previously generated two mouse mutants carrying distinct glia-related mutations in the *PLP1* gene [8] that have initially been identified in patients fulfilling clinical criteria for multiple sclerosis (MS) [9, 10], a neurological CNS disorder known to be related to detrimental neuroinflammation [11, 12]. One of these patients showed features of late-onset progressive MS [9]. The other displayed characteristics of infantile relapsing-remitting MS responding to steroids [10], but whose later course was more typical for complicated hereditary spastic paraplegia type 2 (HSP; Gorman, personal communication), a rare genetically mediated CNS disorder which can be caused by *PLP1* mutations [13]. Indeed, in both mouse mutants, we clearly demonstrated by genetic approaches that the respective disease progression including degeneration of myelin, axon, and neuronal cell bodies is substantially driven by cytotoxic CD8+ T-lymphocytes [8]. These findings suggest that immune modulation might be a promising approach not only to alleviate symptoms of progressive MS but also of some genetic disorders of the CNS, like HSP.

To potentially “translate” our proof-of-principle findings, we here treated the PLP mutant (*PLPmut*) mice with a clinically approved immune modulator, teriflunomide. This treatment led to a substantial alleviation of detrimental neuroinflammation by modulating the composition of CD8+ T-lymphocyte subsets in the CNS towards an expansion of CD8+ CD122+ PD-1+ regulatory T cells resulting in reduced axonal damage. Our studies may therefore have substantial implications for possible treatment approaches not only for progressive forms of MS but also for primarily genetically mediated disorders of the nervous system.

## Methods

### Animals

Mice were kept at the animal facility of the Department of Neurology, University of Wuerzburg, under barrier conditions and at a constant cycle of 12 h in the light (< 300 lx) and 12 h in the dark. All animal experiments were approved by the Government of Lower Franconia, Germany. *PLPmut* (*hPLPG/PlpKo*; *hPLPW/PlpKo*) mice [8] and age-matched wild type (*Wt*) littermates were on a uniform C57BL/6N genetic background. Genotypes were determined by conventional PCR using isolated DNA from ear punch biopsies following previously published protocols [8]. Only hemizygous males or homozygous females were investigated for the present study.

### Immunomodulatory treatment

Teriflunomide (provided by Sanofi Genzyme; Cambridge, USA) was dissolved in autoclaved drinking water containing 0.6% Tween 80 at 60 μg per milligram and provided *ad libitum*. With an approximate consumption of 5 ml per day and 30 g body weight, this corresponds to a dose of 10 mg/kg body weight per day. This concentration is based on previous animal experiments in other laboratories [14, 15] and nearly corresponds to doses used for human multiple sclerosis patients, when a dose conversion scaling is applied [16]. Non-treated controls received autoclaved drinking water without the compounds (but with or without 0.6% Tween 80), and the water with or without the compounds was changed weekly. Water containing only 0.6% Tween 80 had no effect on neuroinflammation and neural damage in *PLPmut* mice (data not shown). Mice were treated for 75, 150, or 330 days (Additional file 1: Figure S1A) and monitored daily regarding defined burden criteria and phenotypic abnormalities. No obvious side effects or significant changes in body weight were detected with the treatment. At the end of the treatment, mice were euthanized with CO_2_ (according to guidelines by the State Office of Health and Social Affairs Berlin), blood was rinsed with phosphate-buffered saline (PBS) containing heparin, and mice were transcardially perfused with 2% paraformaldehyde (PFA) in phosphate-buffered saline (PBS). Tissue was harvested, post-fixed, dehydrated, and processed as described [8].

### Histochemistry and immunofluorescence

Immunohistochemistry was performed on 10-μm-thick longitudinal optic nerve and retina cryo-sections after post-fixation in 4% PFA in PBS or ice-cold acetone for 10 min. Sections were blocked using 5% bovine serum albumin in PBS and incubated over night at 4 °C with one or an appropriate combination of up to three of the following antibodies: rat anti-CD4 (1:1000, Bio-Rad AbD Serotec), rat anti-CD8 (1:500, Bio-Rad AbD Serotec), rat anti-CD11b (1:100, Bio-Rad AbD Serotec), rat anti-CD169 (1:300, Bio-Rad AbD Serotec), mouse anti-SMI32 (1:1000, BioLegend), rat anti-PD-1 (1:100, AbD Serotec), rabbit anti-Ki67 (1:200, abcam), and rat anti-CD8 biotinylated (1:500, BD Biosciences). Immune reactions were visualized using fluorescently labeled (1:300, Dianova) secondary antibodies, streptavidin (1:300, Invitrogen), or biotinylated secondary antibodies (1:100, Vector Laboratories) and streptavidin–biotin–peroxidase (Vector Laboratories, Burlingame, CA) complex using diaminobenzidine–HCl and H_2_O_2_), and nuclei were stained with DAPI (Sigma-Aldrich). Light and fluorescence microscopic images were acquired using an Axiophot 2 microscope (Zeiss) with an attached CCD camera (SPOT Imaging; Diagnostic Instruments, Inc.). Images were minimally processed for generation of figures using Photoshop CS6 (Adobe). For quantification, immunoreactive profiles were counted in at least three non-adjacent optic nerve sections for each animal and related to the area of these sections using the cell counter plugin in ImageJ (National Institutes of Health). For quantification of retinal ganglion cells, the eyes were enucleated and post-fixed in 4% PFA in PBS for 15 min and retinal flat mounts were prepared and air dried overnight. Cresyl violet staining and quantification of Nissl-positive cells in the ganglion cell layer was performed according to previously published protocols in 2–3 images of the middle retinal region per flat mount [8]. SMI32+ RGCs (mostly α-RGCs; [17, 18]) were immunohistochemically labeled in free-floating retinae after post-fixation.

### Flow cytometry of blood leukocytes

Before transcardial perfusion of the euthanized mice, blood was collected from the right atrium of the heart and coagulation was prevented by adding PBS containing heparin. Erythrocytes were lysed and the remaining cells were washed and analyzed by flow cytometry as previously described [19, 20]. Total leukocytes were gated based on forward and side scatter, myeloid cells were stained using PE-conjugated antibodies against CD11b (1:50, BD Biosciences), and T-lymphocytes were stained using antibodies against CD4 and CD8 (1:50, BD Biosciences). At least 1 × 10^5^ leukocytes per mouse were analyzed using a FACSCalibur with CellQuest software (BD Biosciences) and their amount per microliter of blood was calculated.

### Spectral domain optical coherence tomography (OCT)

Mice were subjected to OCT imaging with a commercially available device (Spectralis OCT; Heidelberg Engineering) and additional lenses as previously described [8, 21]. Mice were measured at different ages for longitudinal analysis and the thickness of the innermost retinal composite layer comprising nerve fiber layer (NFL), ganglion cell layer (GCL), and inner plexiform layer (IPL) were measured in high-resolution peripapillary circle scans (at least 10 measurements per scan) by an investigator unaware of the genotype of the mice.

### Accelerating rotarod analysis

Mice were placed on a RotaRod Advanced system (TSE systems), and the time on the constantly accelerating rod (5–50 rpm; max latency 300 s) was measured in five consecutive runs per trial as previously described [8]. Mice were trained with two trials on two consecutive days and measured in a third trial on the third day.

### Experimental design and statistical analysis

**Fig. 1:**
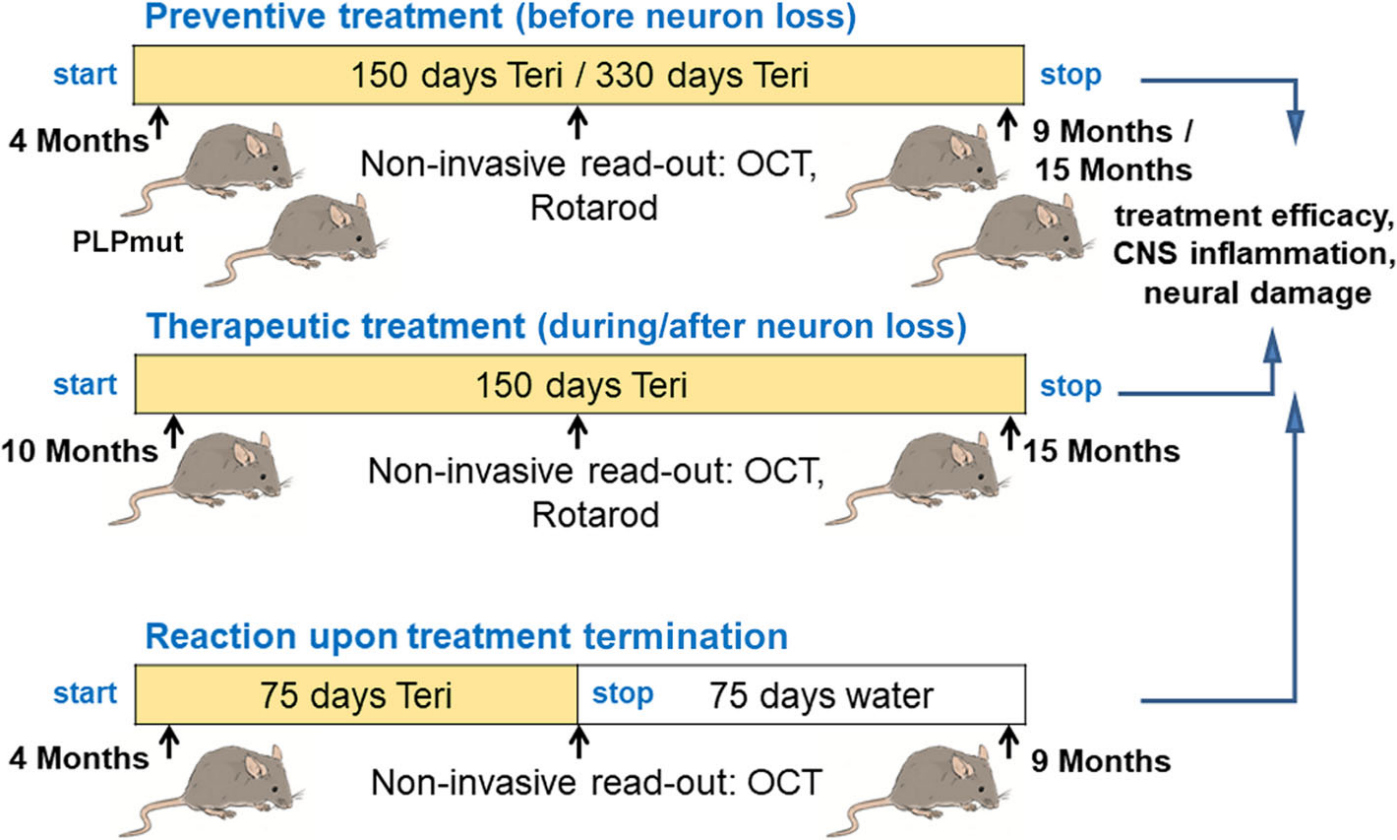
Treatment regimen. Schematic representation of the treatment regimens comprising preventive treatment, therapeutic treatment, and treatment termination, using teriflunomide as medication in the drinking water. “Preventive treatment” started prior to development of major histopathological and clinical features and lasted for 150 or 330 days. “Therapeutic treatment” started when the histopathological phenotype of *PLPmut* mice has already progressed and lasted for 150 days. “Reaction upon treatment termination” was identical as preventive treatment, but treatment was stopped after 75 days, followed by 75 days of survival time lacking treatment. Note non-invasive analysis during treatment allowing longitudinal studies

All quantifications and behavioral analyses were performed by investigators unaware of the genotypes of the respective mice after concealment of genotypes with individual uniquely coded labels. Animals were randomly placed into experimental or control groups (see Fig. 1) according to genotyping results using a random generator (http://www.randomizer.org). For biometrical sample size estimation, the program G*Power (version 3.1.3) was used [22]. Calculation of appropriate sample size groups was performed in a priori power analysis by comparing the mean of two groups with a defined adequate power of 0.8 (1—beta-error) and an α-error of 0.05. To determine the pre-specified effect size *d*, previously published data were considered as comparable reference values [8]. Statistical analysis was performed using PASW Statistics 18 (SPSS, IBM) software. Shapiro-Wilk test was used to check for normal distribution of data. For multiple comparisons, one-way ANOVA followed by Tukey’s post hoc tests (parametric comparison) or Kruskal-Wallis tests with Bonferroni correction (non-parametric comparison) were applied. *P* values considered as significant were indicated by asterisks according to the following scheme: *P < 0.05, **P < 0.01, and ***P < 0.001. Significant differences of a respective treatment group in comparison with wild type mice (*) or untreated *PLPmut* mice (#) are indicated above the corresponding bar. All data are presented as mean +/− standard deviation (SD).

## Results

Based on our previous study identifying neuroinflammation as robust amplifier of two disease-related *PLP1* mutations leading to PLP loss-of-function [8], we aimed to investigate whether the respective inflammatory reactions are treatable with an established immune modulator initially designed for relapsing-remitting multiple sclerosis [23].

We used teriflunomide (10 mg/kg/day) as orally applicable treatment option for the *PLP1* mutant (*PLPmut*) mice and added the compound into the drinking water. Control mice were supplied with normal drinking water lacking the immune modulators or 0.6% Tween-80 only (which had no detectable effects; not shown). Three treatment designs were applied comprising (1) preventive treatment, (2) therapeutic treatment, and (3) treatment termination (Fig. 1). Preventive treatment started prior to development of major histopathological and clinical features (at 4 months of age) and lasted for 150 (“short-term”) or 330 days (“long-term”). Therapeutic treatment started from postnatal month 10 onwards, i.e., when the histopathological phenotype of *PLPmut* mice has already substantially progressed; this treatment also lasted for 150 days. Finally, the regimen “treatment termination” was identical as preventive treatment, but delivery with the compound was stopped after 75 days, followed by analysis after another 75 days of non-treated survival time. With a terminal half-life of 18–37 h and almost complete systemic vanishing within 14 days in mice (EMA assessment report, 529295, 2013), this is a sufficient time frame to exclude sustained pharmacological effects after treatment termination, thus allowing studying putative rebound effects. None of the treatment designs caused any obvious detrimental effects, as revealed by normal body weight and general appearance of the treated mice (not shown).

We first scored the effect of early-onset (preventive) treatment on leukocytes in the peripheral blood (Additional file 1: Figure S1A). Based on flow cytometry, we detected no significant change in the number of leukocytes in peripheral blood in untreated PLP mutants. Teriflunomide did neither reduce T-lymphocyte nor myeloid cell numbers in the circulation (Additional file 1: Figure S1B).

**Fig. 2:**
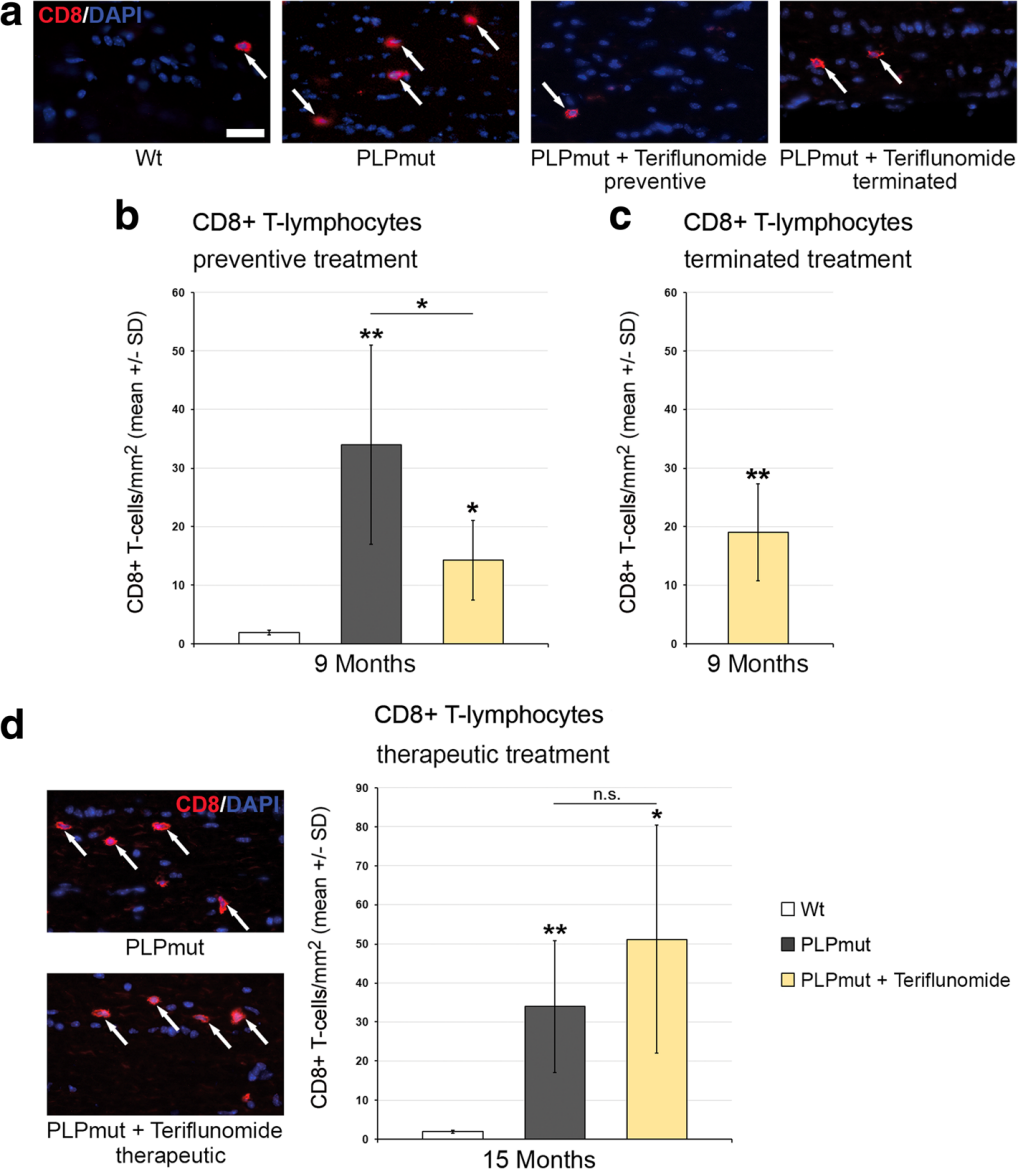
Preventive but not therapeutic treatment impairs the increase of CD8+ T-lymphocyte numbers in *PLPmut* mice. **a** Representative immune fluorescence microscopy of CD8+ T-lymphocytes (arrows) in longitudinal optic nerve sections from *Wt*, untreated mutants (*PLPmut*), preventively treated mutants (150 days, starting from postnatal month 4; PLPmut + teriflunomide preventive), and *PLPmut* mice after treatment interruption at 75 days after treatment onset (PLPmut + teriflunomide terminated). Mice were investigated at 9 months of age. **b** Quantification of CD8+ T-lymphocytes in optic nerve sections of *Wt* and *PLPmut* mice and in *PLPmut* mice after 150 days of preventive treatment. The numbers of CD8+ T cells were significantly increased in the untreated *PLPmut* mice, which was attenuated upon preventive treatment. Mice were investigated at 9 months of age. **c** Quantification of CD8+ T-lymphocytes in optic nerve sections of *PLPmut* mice after 75 days of preventive treatment, followed by 75 days without treatment. Treatment termination did not lead to an overshoot or rebound of T-lymphocyte number, but failed to significantly preserve T-lymphocyte reduction. Mice were investigated at 9 months of age. **d** Immunofluorescent depiction of CD8+ T-lymphocytes (left) and their quantification (right) in optic nerve sections of *Wt* and *PLPmut* mice and in *PLPmut* mice after 150 days of therapeutic treatment (PLPmut + teriflunomide therapeutic) starting at 10 months of age. Therapeutic treatment did not attenuate the increase of CD8+ T-lymphocyte numbers in *PLPmut* mice. Mice were investigated at 15 months of age. Scale bar 30 μm. One-way ANOVA and Tukey’s post hoc tests. *P < 0.05, **P < 0.01. *n* = 5 mice per group

**Fig. 3:**
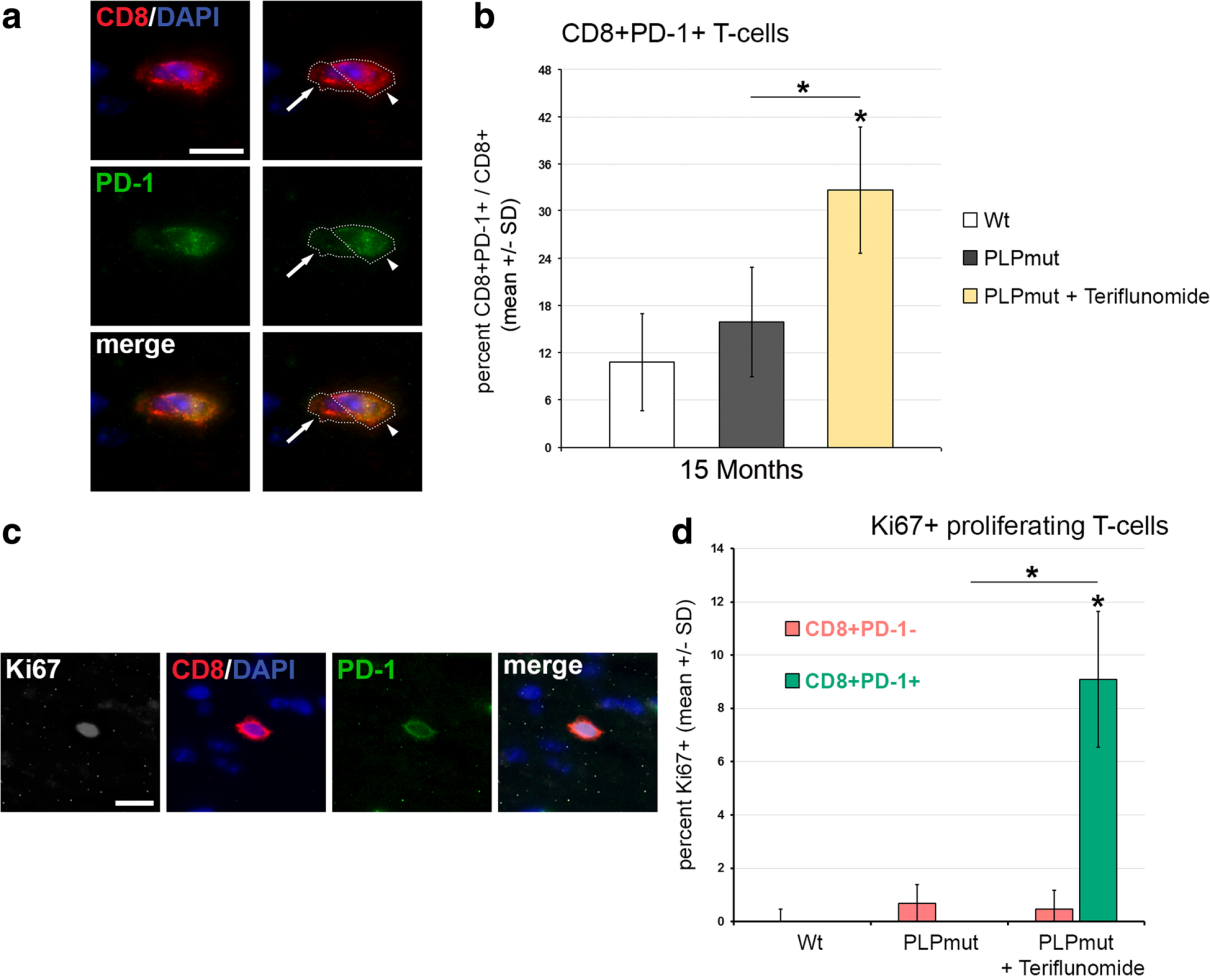
Therapeutic treatment fosters proliferation of regulatory CD8+ PD-1+ T-lymphocytes in optic nerves of *PLPmut* mice. **a** Representative example of the close apposition of a presumed effector T cell (CD8+ PD-1−, arrow) with a regulatory CD8+ PD-1+ T-lymphocyte (arrowhead) in the optic nerve of a 15-month-old therapeutically treated *PLPmut* mouse. **b** Quantification of immunocytochemically labeled CD8+ T cells in optic nerves of 15-month-old *PLPmut* mice revealed a relative increase of CD8+ PD-1+ regulatory T cells upon therapeutic treatment. **c**, **d** Quantitative triple-immunocytochemistry combining antibodies against CD8 (red), against PD-1 (green) and against the proliferation marker Ki67 (gray scale) revealed increased proliferation activity in regulatory CD8+ PD-1+ T-lymphocytes, but not in CD8+ PD-1− presumed effector T cells in optic nerves of therapeutically treated 15-month-old *PLPmut* mice. Scale bars: 10 μm. Kruskal-Wallis test and Bonferroni-Holm correction. *P < 0.05. *n* = 5 mice per group

We, then, quantified the number of CD8+ T-lymphocytes, the likely effector cells in this model [8], in longitudinal sections of the optic nerves. In accordance with our previous studies [8], these cells were elevated in number in the untreated *PLPmut* mice compared with *Wt* mice (Fig. 2). However, upon “short-term” preventive treatment, teriflunomide significantly inhibited the increase of the respective T cell numbers in the CNS (Fig. 2a, b). This inhibitory effect was not significant anymore at 75 days after treatment termination, but an overshoot or rebound by re-occurring T-lymphocytes was not detectable (Fig. 2c). Moreover, therapeutic treatment with teriflunomide failed to reduce the numbers of CD8+ T cells in the CNS. Rather, therapeutic treatment even led to a non-significant trend of elevation of CD8+ T cell numbers (Fig. 2d, e). To correlate the elevated CD8+ T cell numbers with putative therapeutic treatment effects (see below), we considered the possibility that CD8+ T cells might comprise both CD8+ CD122− effector T cells and CD8+ CD122+ regulatory T cells, as previously demonstrated in other models for genetic neurodegenerative diseases [24]. However, while working well in flow cytometry, CD122 antibodies failed to reliably stain cells in immunocytochemistry. An established alternative marker defining CD8 + CD122+ regulatory T cells is PD-1 [25] which worked well in immunocytochemistry and flow cytometry (Fig. 3). To confirm the identity of CD8+ CD122+ as CD8+ PD1+ cells, we used flow cytometry and found that the few CD8+ CD122+ regulatory T cells extracted from the CNS of untreated *PLPmut* mice were indeed PD-1+ while CD8+ CD122− effector T cells were PD-1− (Additional file 1: Figure S2). Interestingly, in optic nerve sections, CD8/PD-1 double labelling revealed a significant shift towards regulatory CD8+ PD-1+ cells in teriflunomide-treated mutants (Fig. 3a, b). The putative regulatory, double-positive cells were occasionally in close apposition with CD8+ PD-1− T cells, as one would expect from regulatory cells inducing cell death of effector T cells (Fig. 3a; [24, 26]. Most interestingly, therapeutic treatment appeared to induce proliferation of CD8+ PD-1+ regulatory T cells in the CNS, as revealed by Ki67 immunocytochemistry (Fig. 3c, d).

**Fig. 4:**
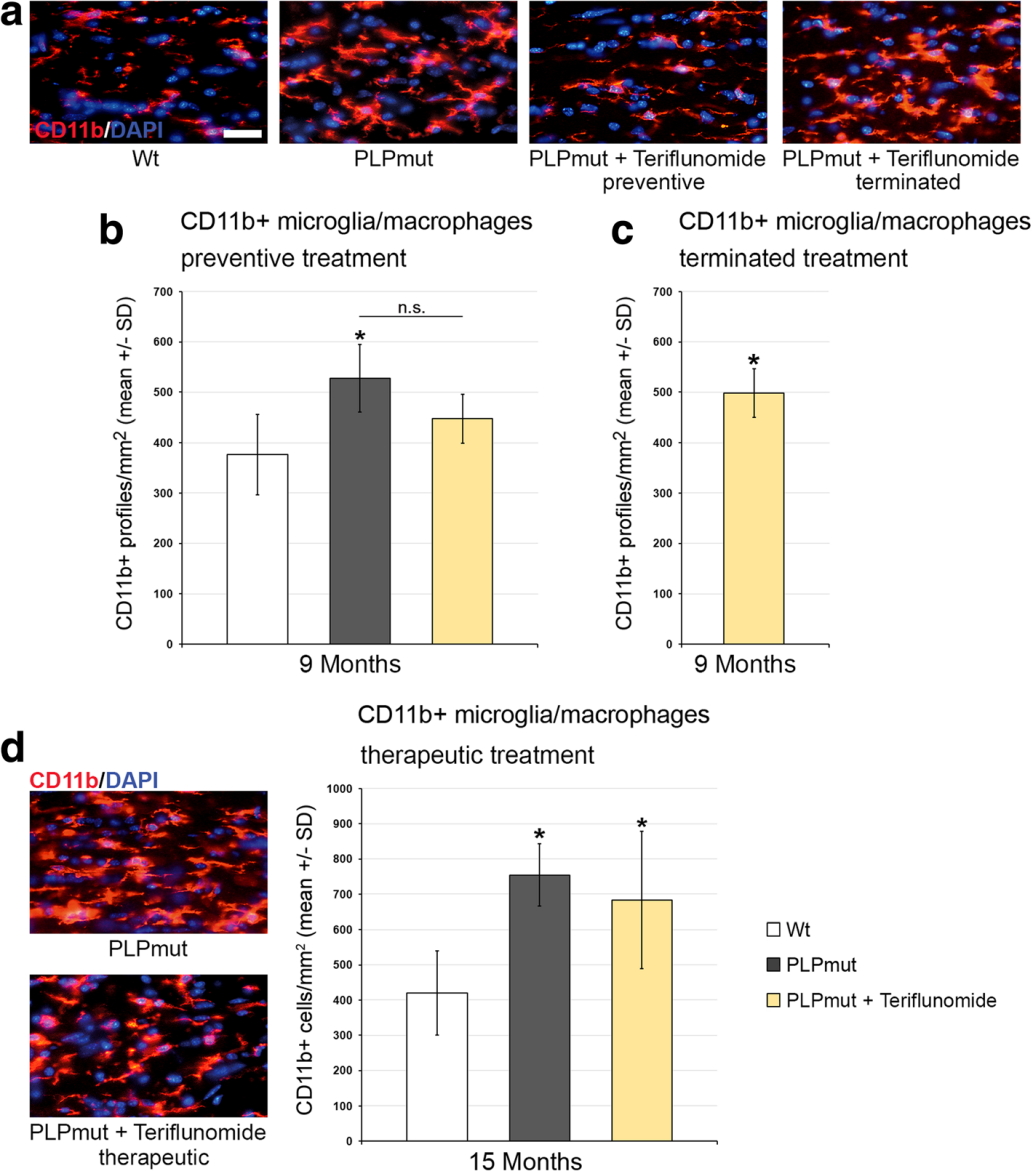
Teriflunomide does not reduce elevated numbers of CD11b+ cells in optic nerves of *PLPmut* mice. **a** Representative immune fluorescence microscopy of CD11b + microglia/macrophages in longitudinal optic nerve sections from *Wt*, untreated mutants (PLPmut), preventively treated mutants (150 days, starting from postnatal month 4; PLPmut + teriflunomide preventive) and *PLPmut* mice after treatment interruption at 75 days after treatment onset (PLPmut + teriflunomide terminated). Mice were investigated at 9 months of age. **b** Quantification of CD11b+ microglia/macrophages in optic nerve sections of *Wt* and *PLPmut* mice and in *PLPmut* mice after 150 days of preventive treatment. The numbers of CD11b+ microglia/macrophages were significantly increased in the untreated *PLPmut* mice. This increase was not significantly reduced upon preventive treatment. Mice were investigated at 9 months of age. **c** Quantification of CD11b+ microglia/macrophages in optic nerve sections of *PLPmut* mice after 75 days of preventive treatment, followed by 75 days without treatment. CD11b+ microglia/macrophages are not reduced in number upon terminated treatment. Mice were investigated at 9 months of age. **d** Immunofluorescent depiction of CD11b+ microglia/macrophages and their quantification (right) in optic nerve sections of *Wt* and *PLPmut* mice and in *PLPmut* mice after 150 days of therapeutic treatment (PLPmut + teriflunomide therapeutic) starting at 10 months of age. Therapeutic treatment did not change the elevated number of CD11b+ microglia/macrophages in *PLPmut* mice. Mice were investigated at 15 months of age. Scale bar 30 μm. One-way ANOVA and Tukey’s post hoc tests. *P < 0.05. *n* = 5 mice per group

**Fig. 5:**
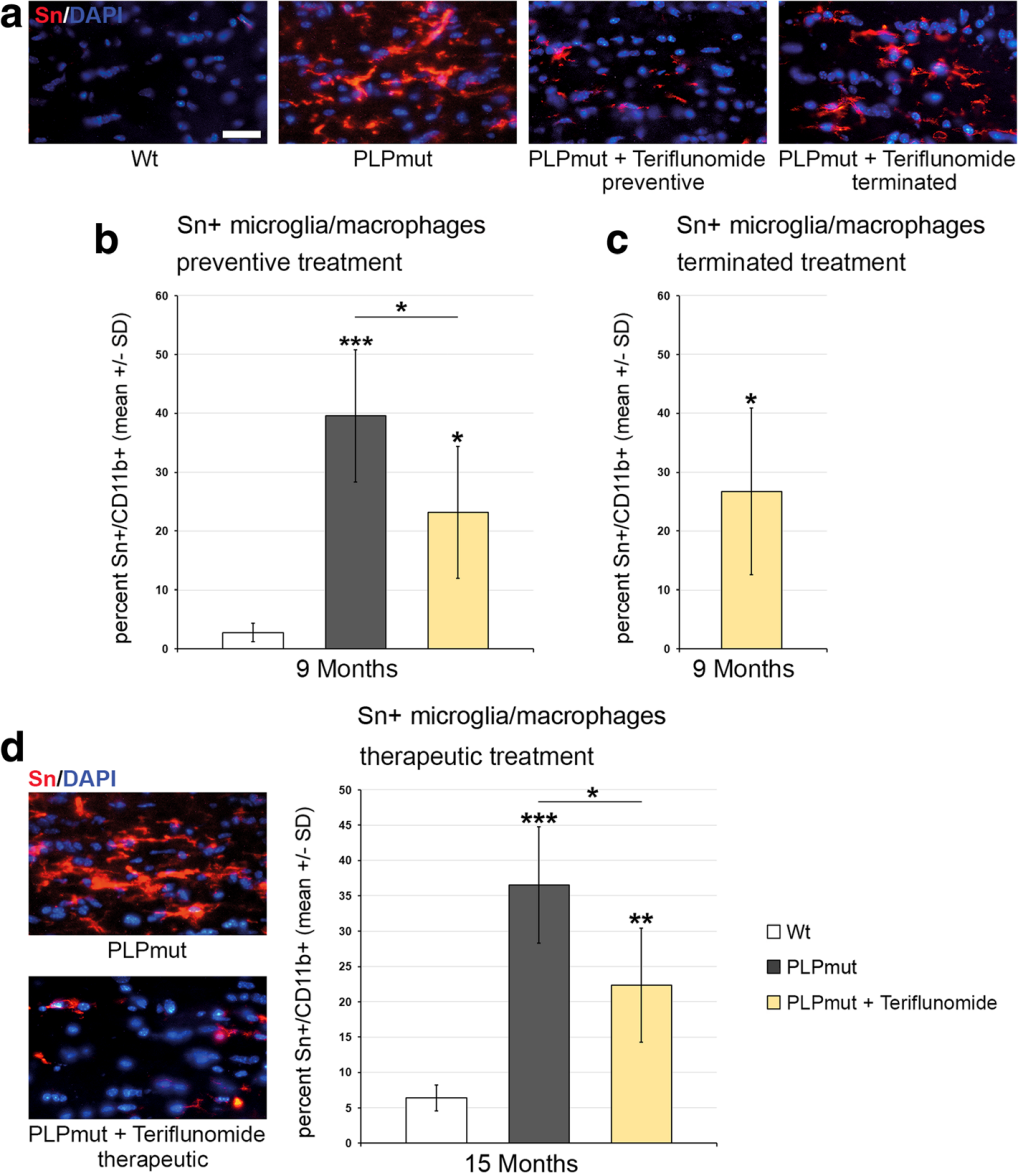
Teriflunomide treatment significantly impairs microglial activation in optic nerves of *PLPmut* mice. **a** Representative immune fluorescence microscopy of Sn+ microglial cells in longitudinal optic nerve sections from *Wt*, untreated mutants (PLPmut), preventively treated mutants (150 days, starting from postnatal month 4; PLPmut + teriflunomide preventive) and *PLPmut* mice after treatment interruption at 75 days after treatment onset (PLPmut + teriflunomide terminated). Mice were investigated at 9 months of age. **b** Quantification of Sn + microglial cells in optic nerve sections of *Wt* and *PLPmut* mice and in *PLPmut* mice after 150 days of preventive treatment. The numbers of Sn + activated microglial cells were significantly increased in the untreated *PLPmut* mice, which was partially forstalled upon preventive treatment. Mice were investigated at 9 months of age. **c** Quantification of Sn + microglial cells in optic nerve sections of *PLPmut* mice after 75 days of preventive treatment, followed by 75 days without treatment. Reduced elevation of Sn+ microglial cells after terminated treatment was not significant anymore. Mice were investigated at 9 months of age. **d** Immunofluorescent depiction of Sn+ microglial cells and their quantification (right) in optic nerve sections of *Wt* and *PLPmut* mice and in *PLPmut* mice after 150 days of therapeutic treatment (PLPmut + teriflunomide therapeutic) starting at 10 months of age. Therapeutic treatment significantly reduced the elevation of Sn+ microglial cell numbers in *PLPmut* mice. Mice were investigated at 15 months of age. Scale bar, 30 μm. One-way ANOVA and Tukey’s post hoc tests. *P < 0.05, **P < 0.01, ***P < 0.001. *n* = 5 mice per group

Teriflunomide treatment had similar, cell number-reducing effects on CD4+ T cells, when preventive, but not therapeutic treatment was applied (Additional file 1: Figure S3). The reduced numbers of CD4+ T cells were not maintained after treatment termination (Additional file 1: Figure S3B). With regard of CD11b+ microglial/macrophage-like cell numbers, the treatment led to a reduction when preventive treatment was applied (Fig. 4a, b), but this reduction was not persistent upon treatment termination (Fig. 4c). Therapeutic treatment failed to reduce microglial cell numbers (Fig. 4d). However, the numbers of activated sialoadhesin (Sn)+ microglial cells were significantly reduced by both preventive and therapeutic treatment with teriflunomide (Fig. 5).

**Fig. 6:**
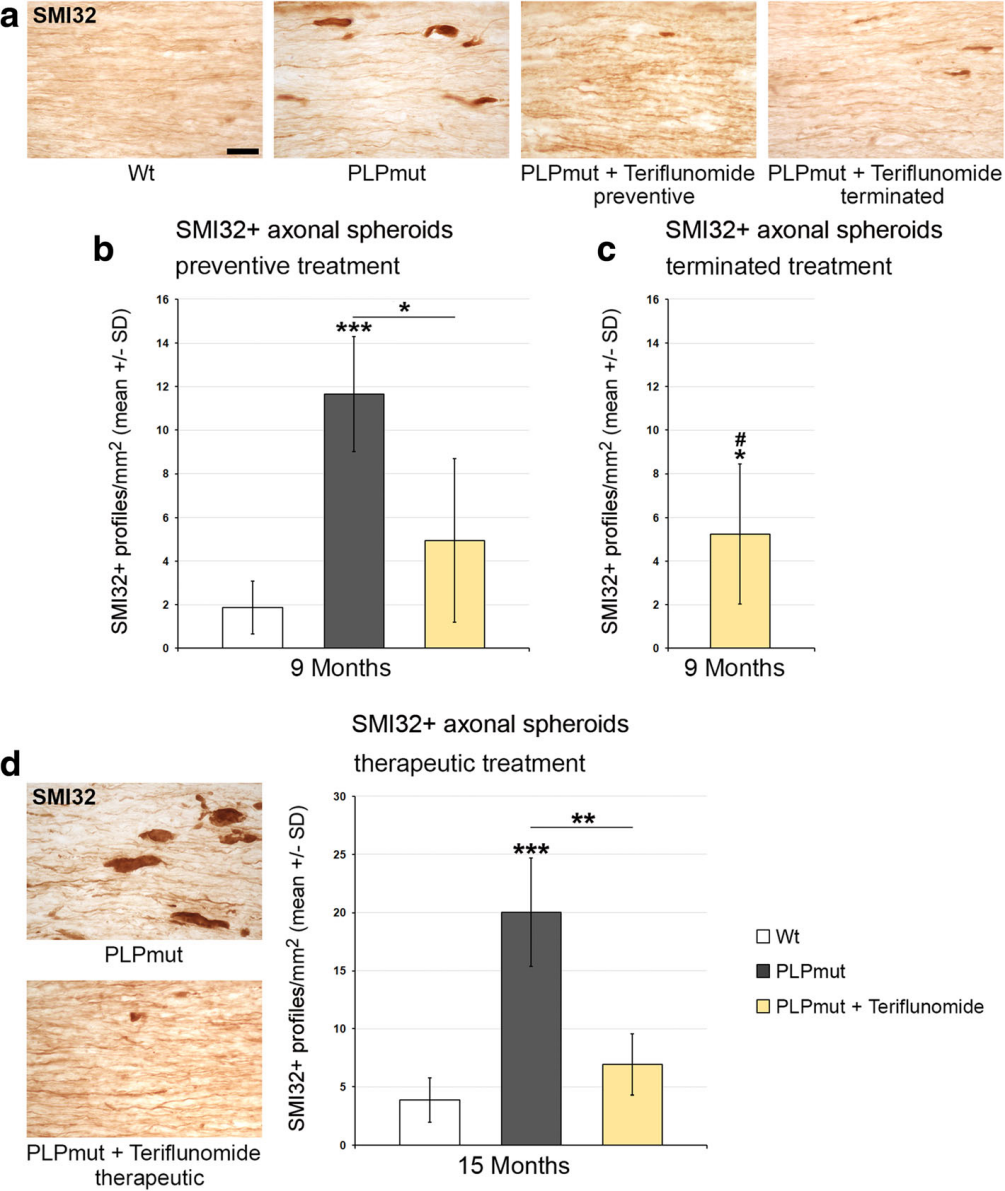
Teriflunomide treatment significantly reduces axonopathic alterations in optic nerves of *PLPmut* mice. **a** Representative immunocytochemical identification of SMI32+ axonal spheroids in longitudinal optic nerve sections from *Wt*, untreated mutants (PLPmut), preventively treated mutants (150 days, starting from postnatal month 4; PLPmut + teriflunomide preventive), and *PLPmut* mice after treatment interruption at 75 days after treatment onset (PLPmut + teriflunomide terminated). Mice were investigated at 9 months of age. **b** Quantification of SMI32+ axonal spheroids of *Wt* and *PLPmut* mice and of *PLPmut* mice after 150 days of preventive treatment. The numbers of SMI32+ axonal spheroids were robustly increased in the untreated *PLPmut* mice, but this increase was significantly attenuated upon preventive treatment. Mice were investigated at 9 months of age. **c** Quantification of SMI32+ axonal spheroids in optic nerve sections of *PLPmut* mice after 75 days of preventive treatment, followed by 75 days without treatment. Reduction of SMI32+ axonal spheroid numbers persisted after terminated treatment. Mice were investigated at 9 months of age. **d** Immunocytochemical depiction of SMI32+ axonal spheroids (left) and their quantification (right) in optic nerve sections of *Wt* and *PLPmut* mice and in *PLPmut* mice after 150 days of therapeutic treatment (PLPmut + teriflunomide therapeutic) starting at 10 months of age. Therapeutic treatment significantly attenuated elevation of SMI32+ axonal spheroid numbers in *PLPmut* mice. Mice were investigated at 15 months of age. Scale bar, 30 μm. One-way ANOVA and Tukey’s post hoc tests. *^,#^*P* < 0.05, **P < 0.01, ***P < 0.001. *n* = 5 mice per group

**Fig. 7:**
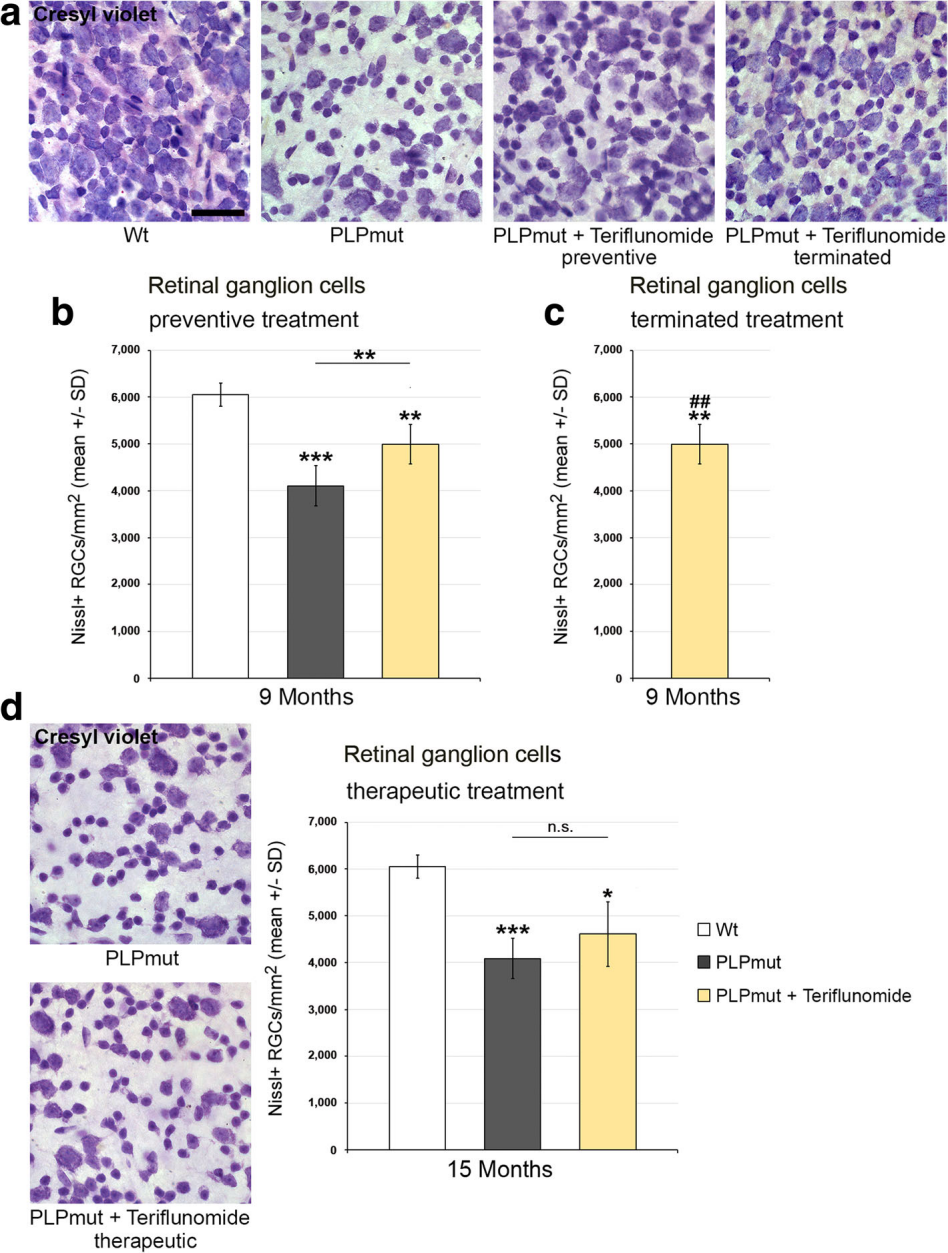
Teriflunomide treatment prevents but does not reverse retinal ganglion cell loss in *PLPmut* mice. **a** Cresyl violet-labeled perikarya in the ganglion cell layer of flat-mounted retinae from *Wt*, untreated mutants (PLPmut), preventively treated mutants (150 days, starting from postnatal month 4; PLPmut + teriflunomide preventive) and *PLPmut* mice after treatment interruption at 75 days after treatment onset (PLPmut + teriflunomide terminated). Mice were investigated at 9 months of age. **b** Quantification of cresyl violet-labeled perikarya in the ganglion cell layer of flat-mounted retinae of *Wt* and *PLPmut* mice and of *PLPmut* mice after 150 days of preventive treatment. The numbers of cresyl violet-labeled perikarya were significantly reduced in the untreated *PLPmut* mice, but significantly rescued upon preventive treatment. Mice were investigated at 9 months of age. **c** Quantification of cresyl violet-labeled perikarya in the ganglion cell layer of flat-mounted retinae of *PLPmut* mice after 75 days of preventive treatment, followed by 75 days without treatment. Rescue of cresyl violet-labeled perikarya persisted after treatment termination. Mice were investigated at 9 months of age. **d** Depiction of cresyl violet-labeled perikarya in the ganglion cell layer (left) and their quantification (right) in *Wt* and *PLPmut* mice and in *PLPmut* mice after 150 days of therapeutic treatment (PLPmut + teriflunomide therapeutic) starting at 10 months of age. Therapeutic treatment did not significantly reverse retinal ganglion cell loss in *PLPmut* mice. Mice were investigated at 15 months of age. Scale bar, 50 μm. One-way ANOVA and Tukey’s post hoc tests. *P < 0.05, **^,##^*P* < 0.01, ***P < 0.001. *n* = 5 mice per group

Regarding histopathological changes, we first scored SMI32+ axonal spheroids in longitudinal sections of optic nerves (Fig. 6). Preventive treatment reduced the number of these profiles and this reduction persisted when treatment was terminated after the first 75 days. Interestingly, teriflunomide treatment also had a robust effect on the number of axonal spheroids upon therapeutic treatment. Similar beneficial treatment effects were seen when retinal ganglion cell numbers were determined, with the exception that therapeutic treatment failed to show a significant effect (Fig. 7). These combined findings on the therapeutic treatment regimen suggest that ongoing axonal perturbation can be attenuated with teriflunomide, but neurons having degenerated during the first 10 months of age cannot be restored. This may explain a lack of clinical improvement upon therapeutic treatment as presented below (Fig. 11b).

**Fig. 8:**
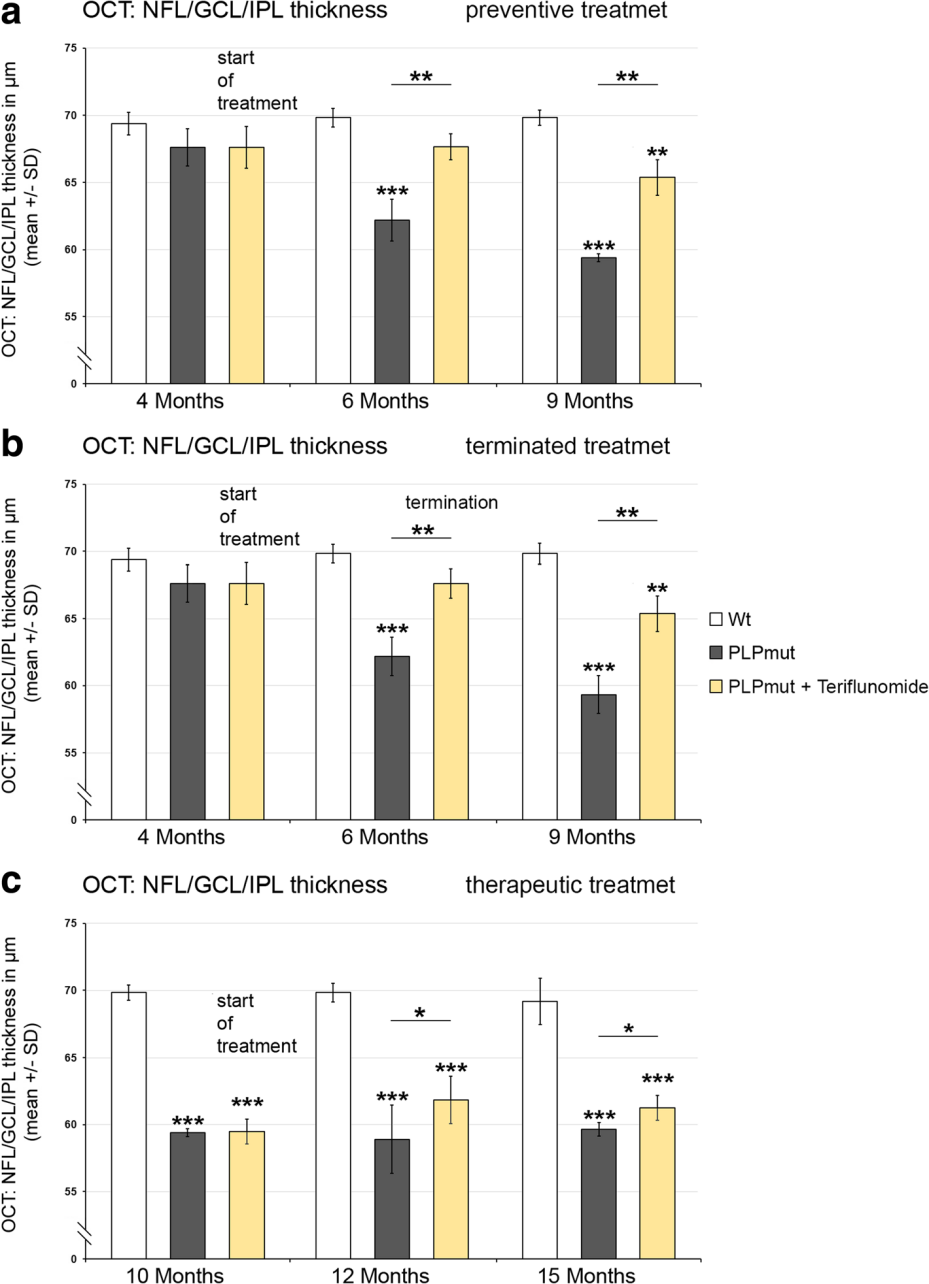
OCT reveals reduced thinning of the retinal NFL/GCL/IPL composite layer upon preventive and terminated treatments and regenerative thickening upon therapeutic treatment. **a** Longitudinal analysis of NFL/GCL/IPL thickness of *Wt*, untreated *PLPmut* mice, and preventively treated *PLPmut* mice at 4, 6, and 9 months of age. Note reduced thinning of the retinal NFL/GCL/IPL composite layer. **b** Longitudinal analysis of NFL/GCL/IPL thickness of *Wt*, untreated *PLPmut* mice and *PLPmut* mice after 75 days of preventive treatment, followed by 75 days without treatment. Mice were investigated at 4, 6, and 9 months of age. Note that improved NFL/GCL/IPL composite layer thickness was preserved even after treatment termination. **c** Longitudinal analysis of NFL/GCL/IPL thickness of *Wt*, untreated *PLPmut* mice, and therapeutically treated *PLPmut* mice. Mice were investigated at 10, 12, and 15 months of age. Note unexpected “regenerative” thickening of NFL/GCL/IPL composite layer as a reaction upon therapeutic treatment at 12 and 15 months. One-way ANOVA and Tukey’s post hoc tests. *P < 0.05, **P < 0.01, ***P < 0.001. *n* = 5 mice per group

**Fig. 9:**
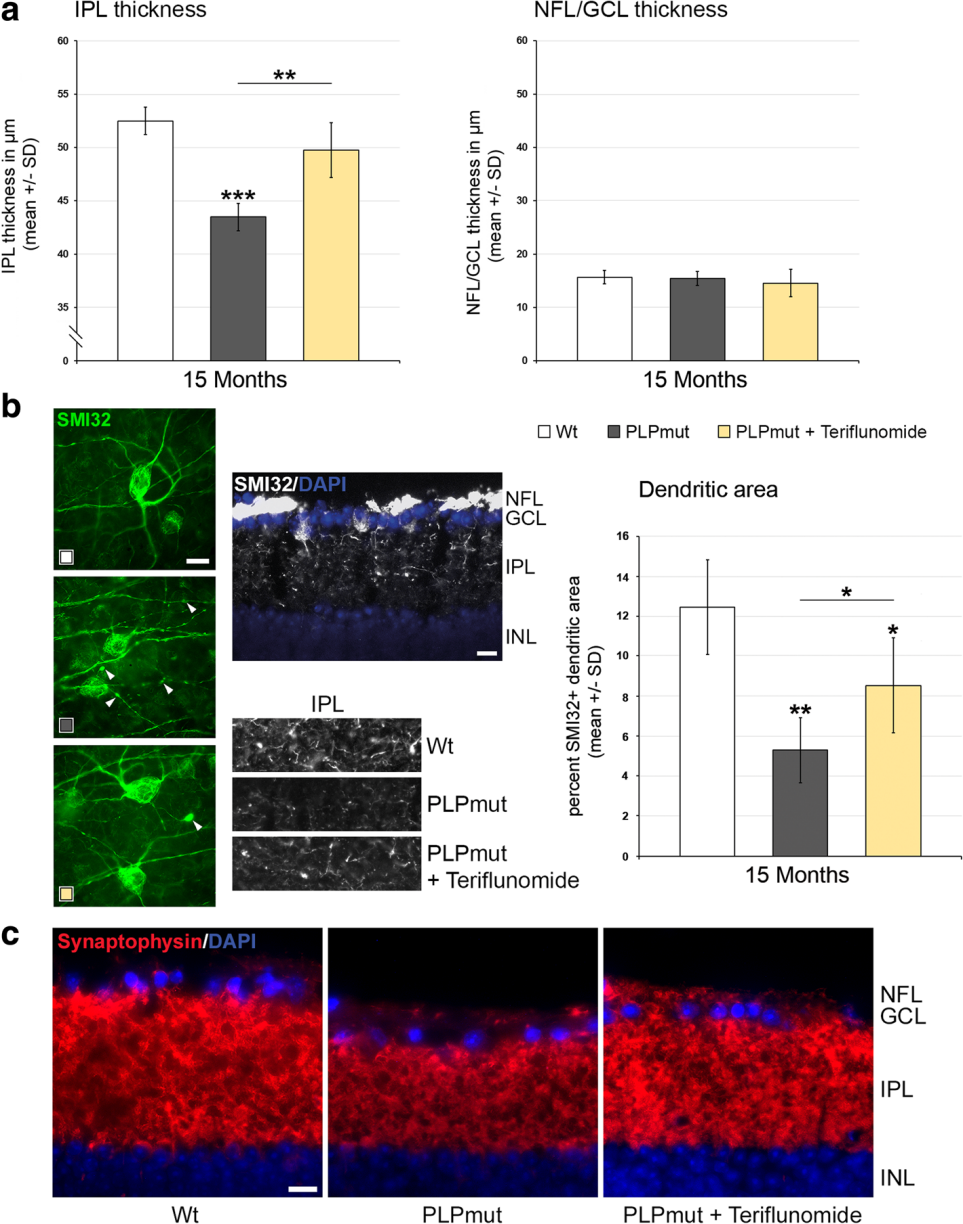
The regenerative thickening of the innermost retinal composite layer upon therapeutic treatment is driven by restoration of dendritic arborization within the IPL. **a** Histological quantification of thickness of the IPL (left) and NFL/GCL (right) in *Wt*, *PLPmut*, and *PLPmut* mice after therapeutic treatment identifies the IPL as the responsible layer for the “regenerative” thickening. **b** Left, flat mount preparations of retinae. SMI32+ retinal ganglion cells display robust dendritic arborization in *Wt* mice (white icon) while the same cell types of untreated *PLPmut* mice (dark-gray icon) show “fragile” arbors of reduced extension and abundant degenerative varicosities (arrowheads). Therapeutic treatment (yellow icon) partially restored dendritic arborization and reduced numbers of degenerative varicosities. Middle, top: SMI32 immunofluorescence on a transverse section of the retina of a *Wt* mouse. A subpopulation of retinal ganglion cells in the GCL, their fasciculating axons in the NFL and their dendritic trees protruding into the IPL are visible. Middle, below: SMI32 immunofluorescence on transverse sections of the IPLs of *Wt*, *PLPmut*, and therapeutically treated *PLPmut* mice. Note “re-appearance” of SMI32-immunoreactivity reflecting regenerative dendritic trees in the IPL of the treated *PLPmut* mice. Right, quantification of SMI32+ dendritic areas in *Wt*, *PLPmut*, and therapeutically treated *PLPmut* mice. **c** Synaptophysin immunoreactivity in the IPLs of *Wt*, untreated and therapeutically treated *PLPmut* mice support the view that “regenerative” thickening of the IPL upon treatment is related to restoration of dendritic trees (as shown in **a**, **b**) and of their corresponding presynaptic terminals. Scale bars, 10 μm. One-way ANOVA and Tukey’s post hoc tests. *P < 0.05, **P < 0.01, ***P < 0.001. *n* = 5 mice per group

Using a non-invasive technique, optical coherence tomography, we were also able to analyze living, treated, and non-treated individuals longitudinally, reflecting the translational character of this part of the study. Recapitulating our previous findings [8], there was a progredient, significant thinning of the NFL/GCL/IPL composite layer in *PLPmut* mice (Fig. 8), whereas the outer retinal layers were not affected (not shown). Of note, at 4 months of age, the start of treatment in preventive and terminated regimens, there was already a mild, non-significant tendency towards reduction of the inner composite layer thickness detectable (Fig. 8a, b). Interestingly, preventive treatment with teriflunomide lead to a slowing of thinning of the NFL/GCL/IPL composite layer (Fig. 8a). When treatment was interrupted after 75 days, an improved composite layer thickness was still detectable after another 75 days without treatment, when compared to *PLPmut* mice which were never treated (Fig. 8b). Therapeutic treatment starting at 10 months of age lead to an unexpected “regenerative” thickening of the composite layers in teriflunomide-treated mutants after 2 months. This increased composite layer thickness was maintained for another 3 months in teriflunomide-treated mutants (Fig. 8c). To characterize the “regenerative” thickening of the inner composite layers as result of therapeutic treatment in more detail, we first determined which of the three individual layers (NFL/GCL/IPL) was responsible for this phenomenon in 15-month-old teriflunomide-treated mutants. We identified that the IPL rather than NFL/GCL was responsible for this phenomenon (Fig. 9a). As this layer contains the dendritic trees of retinal ganglion neurons, we labeled a subpopulation of these neurons with SMI32 antibodies against non-phosphorylated neurofilaments. In *Wt* mice, prominent SMI32 staining of fasciculating, (at this level) unmyelinated axons of retinal ganglion neurons in the NFL was underlied by the SMI32+ perikarya of some neurons in the GCL, the dendrites of which protruding into the IPL (Fig. 9b). The immunofluorescent signal of these dendritic trees in the IPL was strongly reduced in 15 months old, untreated *PLPmut* mice, but partially “re-appeared” in mutants receiving teriflunomide as therapeutic treatment. These findings were supported by quantifying the area occupied by the SMI32+ dendrites (Fig. 9b) and by scoring synaptophysin immunoreactivity in the IPLs of *Wt*, untreated and therapeutically teriflunomide-treated mutants (Fig. 9c).

As it is conceivable that some of the therapeutic treatment effects could be explained by direct neuroprotective or regeneration-promoting effects of teriflunomide, we investigated this possibility by therapeutically treating *Rag1*-deficient *PLPmut* mice lacking mature T- and B-lymphocytes (Additional file 1: Figures S4 and S5). As previously shown, the untreated lymphocyte-depleted mutants show a partial rescue of the histopathological phenotype [8]. This partial rescue could not be further improved by therapeutic treatment with teriflunomide, when number of microglial cells (Additional file 1: Figure S4) or histopathological criteria (Additional file 1: Figure S5) were applied. These findings fail to provide evidence for a direct neuroprotective effect of teriflunomide independent of modulating adaptive immune reactions in the present disease model.

**Fig. 10:**
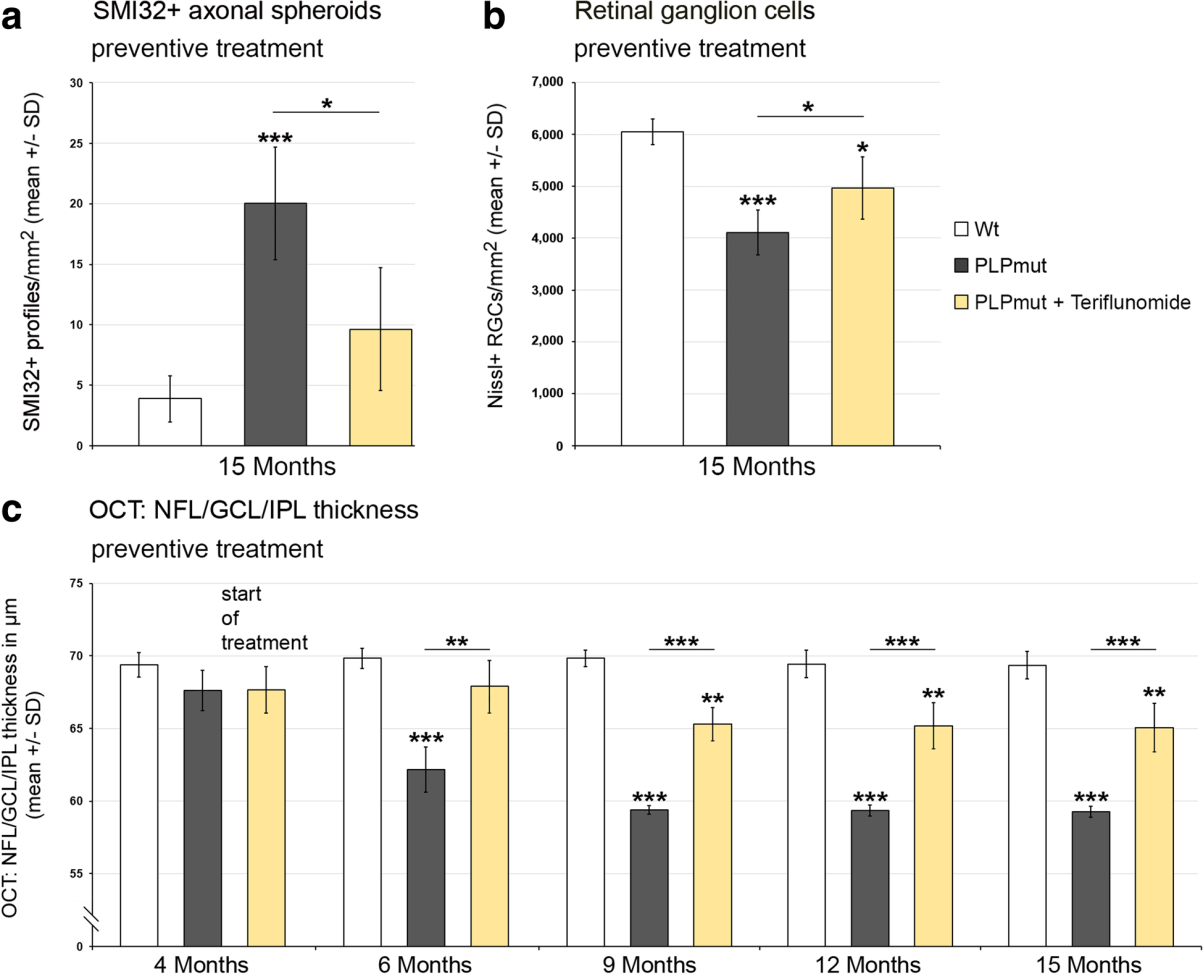
Long-term preventive treatment improves axonal and neuronal degeneration in the visual system and leads to sustained preservation of the NFL/GCL/IPL composite layer of the retina. **a** Quantification of SMI32+ axonal spheroids of *Wt* and *PLPmut* mice and of *PLPmut* mice after 330 days of preventive treatment. The numbers of SMI32+ axonal spheroids were significantly increased in the untreated *PLPmut* mice, but elevation was significantly attenuated upon long-term preventive treatment. Mice were investigated at 15 months of age. **b** Quantification of perikarya in the ganglion cell layer of flat-mounted retinae of *Wt* and *PLPmut* mice and of *PLPmut* mice after 330 days of preventive treatment. The numbers of perikarya were significantly reduced in the untreated *PLPmut* mice, but partially rescued upon long-term preventive treatment. Mice were investigated at 15 months of age. **c** Longitudinal analysis of NFL/GCL/IPL thickness of *Wt*, untreated *PLPmut* mice and long-term preventively treated *PLPmut* mice at 4, 6, 9, 12, and 15 months of age by OCT. Note reduced thinning of the retinal NFL/GCL/IPL composite layer over the complete period of teriflunomide treatment. One-way ANOVA and Tukey’s post hoc tests. *P < 0.05, **P < 0.01, ***P < 0.001. *n* = 5 mice per group

**Fig. 11:**
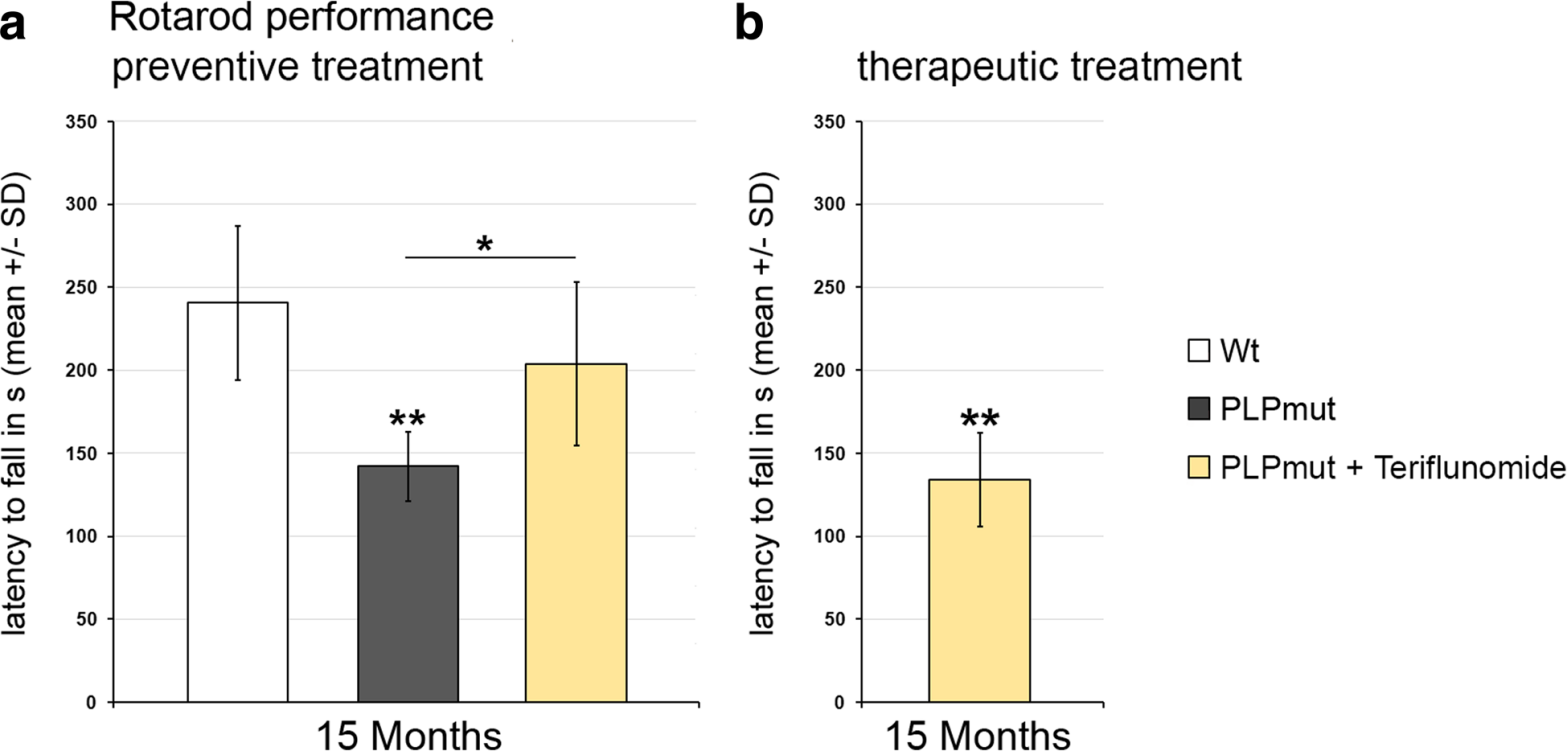
Preventive but not therapeutic treatment improves clinical outcome in *PLPmut* mice. **a** Accelerating rotarod performance of *Wt*, *PLPmut*, and *PLPmut* mice which received long-term preventive treatment. Treatment significantly improved latency to fall. **b** Accelerating rotarod performance of *PLPmut* mice which received therapeutic treatment for 5 months. Treatment failed to significantly improve latency to fall, most likely due to already progressed neurodegeneration at 10 months of age, when therapeutic treatment started. One-way ANOVA and Tukey’s post hoc tests. *P < 0.05, **P < 0.01. *n* = 5 mice per group

As teriflunomide showed robust treatment effects in experiments lasting for 5 months, we performed a long-term, preventive treatment approach lasting nearly a year. We found that upon long-term treatment, the numbers of T-lymphocytes and the total number of microglial cells reached the level of non-treated mutants (Additional file 1: Figure S6). However, similar as in the therapeutic treatment approach (Fig. 3), the percentage of CD8+ PD-1+ putative regulatory T cells was increased after long-term treatment (35.56 ±  8.24%) in comparison to untreated *PLPmut* mice (16.47 ±  5.64%). Moreover, the numbers of activated Sn+ microglial cells were still reduced after long-term treatment conditions (Additional file 1: Figure S6). In line with our findings related to treatment duration of 5 months, long-term preventive treatment with teriflunomide reduced the numbers of SMI32+ axonal spheroids (Fig. 10a) and partially prevented retinal ganglion cell degeneration (Fig. 10b). Of note, longitudinal studies using non-invasive OCT document the long-term preservation of the inner composite layer of the retina (Fig. 10c), reflecting the constant efficacy of the treatment. This was in line with improved latencies to fall in rotarod test upon preventive long-term treatment demonstrating that the treatment had clinically relevant effects (Fig. 11a). In contrast, therapeutic treatment for 5 months failed to improve clinical outcome (Fig. 11b), most likely due to already progressed neurodegeneration at 10 months of age, when therapeutic treatment started.

## Discussion

We show that pathogenetically relevant secondary inflammation aggravating a myelin-related genetic defect [8] can substantially be alleviated in its histopathological impact by the clinically approved immune modulator teriflunomide. The treatment was well tolerated and highly efficient when given along a preventive design and was even able to reduce histopathological features when therapeutically applied late after disease onset. Additionally, in case of treatment interruption, the compound did not cause overshooting rebound reactions; instead, preserved beneficial histopathological consequences were detectable even 75 days after treatment termination.

After short-term preventive treatment with teriflunomide, a reduction in the number of T-lymphocytes was observed in the CNS, an observation being in line with the cytostatic impact of the compound on activated adaptive immune cells [27, 28]. Such a reduction in adaptive immune cells in the CNS was not observed in PLP mutants after long-term preventive treatment or in mutants therapeutically treated with teriflunomide at 10 months of age, which, at first glance, appeared surprising, as there was a concomitant substantial amelioration of histopathological features. In fact, we identified teriflunomide acting by shifting regulatory versus effector CD8+ T cells in the CNS of the treated mice explaining the beneficial therapeutic treatment effect in the presence of a high number of CD8+ T-lymphocytes. Interestingly, teriflunomide has previously been identified as shifting Th1 and Th2 reactions in favor of the latter [29]. Here, we extend this model by implicating CD8+ CD122- PD-1− cytotoxic effector T cells and CD8+ CD122+ PD-1+ regulatory T cells. In this context, it is interesting that microglial Sn has previously been identified as an effective inhibitor of regulatory CD8+ CD122+ T cells in the diseased CNS [24] and that teriflunomide reduced the number of Sn+ microglial cells (this study). However, as teriflunomide treatment could not further reduce Sn expression in *PLPmut*/*Rag1*^−/−^ mice (see above), we propose that the compound acts on adaptive immune cells, which reciprocally influence Sn expression on microglia/macrophages. A possible mechanism explaining our findings might be the inhibitory effect of teriflunomide on the interaction of T cells with antigen presenting cells [30], which—in the present model—could be identical with activated Sn + microglia/macrophages.

While increased regulatory T cell proliferation might appear paradox under a cytostatic drug therapy, it is known that teriflunomide has no effect on proliferation of lymphocytes that do not require de novo pyrimidine synthesis to self-renew, like resting lymphocytes [31]. It has recently been shown that regulatory CD4+ FoxP3^high^ T cells show resilience towards restriction of glutamine-dependent purine and pyrimidine syntheses [32]. Moreover, results from the Teri-DYNAMIC study showed that teriflunomide also increased regulatory T cell counts and reduced Th1 counts and clonal diversity of CD4+ T cells in RRMS patients. Arguing for an immunomodulatory rather than immunosuppressive mechanism of action, T cells from patients treated with teriflunomide were still able to mount appropriate proliferative and cytokine responses [33]. It remains to be determined if similar resilience mechanisms might explain the unaffected proliferation capacity of the CD8+ CD122+ PD-1+ regulatory T cell population in the *PLPmut* model undergoing teriflunomide treatment.

The purpose of our present study was to investigate whether “genetic” models sharing pathogenetic pathways with progressive MS can efficiently be treated with established immune modulators. As opposed to the treatable relapsing MS, the progressive subforms are often thought to occur independently of inflammation, as they are usually poorly responsive to conventional immune modulatory therapeutic approaches [34, 35]. Here, we show that the established immune modulator teriflunomide substantially improves histopathology and clinical deficits when applied preventively. In addition, teriflunomide succeeded to ameliorate disease outcome when given relatively late during pathogenesis. While there was no regeneration of lost neurons after therapeutic treatment, ongoing axonal damage was halted and inner retinal thickness most likely reflecting dendritic arborization was recovered by remaining neurons. This therapeutic effect demonstrates some endogenous capacity for regeneration which is normally blocked by inflammation but allowed or facilitated by the therapy-induced proliferation of parenchymal regulatory CD8+ CD122+ PD-1+ T-lymphocytes which control cytotoxic CD8+ effector T cells [24]. While this is to our knowledge a novel immune-modulating mechanism of teriflunomide, none of the previously described multiple non-immune effects of the drug [28] appear to play a major role in our disease model, as revealed by lack of further neuropathological improvement in teriflunomide-treated *PLPmut*/*Rag1*^−/−^ mice.

## Conclusions

All in all, our study is encouraging to consider teriflunomide for mitigating or modulating chronic detrimental neuroinflammation in progressive MS in humans. These results support ongoing clinical trials with teriflunomide showing stabilization or improvement of long-term disability in most MS patients regardless of subtype [36, 37]. Since our model not only shares features with progressive MS but also comprises features of other immune-related leukodystrophies or hereditary spastic paraplegia [4, 8, 38–40], it is plausible to assume that the immune modulators may also alleviate some forms of these diseases. A recent study from our laboratory showed that not only teriflunomide but also the immune modulatory sphingosine-1-phosphate analogue fingolimod substantially alleviated axonal damage, neuron loss and retinal thinning in two models for the fatal, infantile and juvenile inherited storage disorders, CLN1 and CLN3 [20]. These findings may not only give hope to patients suffering from inflammation-related orphan diseases with no causal treatment, but may additionally exemplify that neuroinflammation may be a therapeutically relevant common disease pathway of many primarily unrelated genetic disorders of the nervous system [4].

## Additional file

Additional file 1: **Figure S1.** Lack of impact of teriflunomide treatment on blood leukocyte numbers. **Figure S2.** Confirmation of PD-1 expression as a marker of CD8+ CD122+ regulatory T cells in the CNS of *PLPmut* mice. **Figure S3.** Preventive but neither terminated nor therapeutic treatment impairs the increase of CD4+ T-lymphocyte numbers in *PLPmut* mice. **Figure S4.** Lack of evidence for an immune-unrelated effect of therapeutic teriflunomide treatment. **Figure S5.** Lack of evidence for an immune-unrelated effect of therapeutic teriflunomide treatment on histopathological features. **Figure S6.** Long-term preventive treatment does not reduce T-lymphocyte and microglia/macrophage numbers but leads to a reduction of activated Sn+ microglial cells. (PDF 1034 kb)

## Abbreviations

OCT: Optical coherence tomography
PMS: Progressive multiple sclerosis
RGC: Retinal ganglion cell

## References

[1] Nave KA (2010). Myelination and the trophic support of long axons. Nat Rev Neurosci.

[2] Nave KA (2010). Myelination and support of axonal integrity by glia. Nature.

[3] Luders KA, Patzig J, Simons M, Nave KA, Werner HB (2017). Genetic dissection of oligodendroglial and neuronal Plp1 function in a novel mouse model of spastic paraplegia type 2. Glia.

[4] Groh J, Martini R (2017). Neuroinflammation as modifier of genetically caused neurological disorders of the central nervous system: understanding pathogenesis and chances for treatment. Glia.

[5] Funfschilling U, Supplie LM, Mahad D, Boretius S, Saab AS, Edgar J, Brinkmann BG, Kassmann CM, Tzvetanova ID, Mobius W (2012). Glycolytic oligodendrocytes maintain myelin and long-term axonal integrity. Nature.

[6] Ip CW, Kroner A, Bendszus M, Leder C, Kobsar I, Fischer S, Wiendl H, Nave KA, Martini R (2006). Immune cells contribute to myelin degeneration and axonopathic changes in mice overexpressing proteolipid protein in oligodendrocytes. J Neurosci.

[7] Ip CW, Kroner A, Groh J, Huber M, Klein D, Spahn I, Diem R, Williams SK, Nave KA, Edgar JM, Martini R (2012). Neuroinflammation by cytotoxic T-lymphocytes impairs retrograde axonal transport in an oligodendrocyte mutant mouse. PLoS One.

[8] Groh J, Friedman HC, Orel N, Ip CW, Fischer S, Spahn I, Schaffner E, Horner M, Stadler D, Buttmann M (2016). Pathogenic inflammation in the CNS of mice carrying human PLP1 mutations. Hum Mol Genet.

[9] Warshawsky I, Rudick RA, Staugaitis SM, Natowicz MR (2005). Primary progressive multiple sclerosis as a phenotype of a PLP1 gene mutation. Ann Neurol.

[10] Gorman MP, Golomb MR, Walsh LE, Hobson GM, Garbern JY, Kinkel RP, Darras BT, Urion DK, Eksioglu YZ (2007). Steroid-responsive neurologic relapses in a child with a proteolipid protein-1 mutation. Neurology.

[11] Kawachi I, Lassmann H (2017). Neurodegeneration in multiple sclerosis and neuromyelitis optica. J Neurol Neurosurg Psychiatry.

[12] Winkelmann A, Loebermann M, Reisinger EC, Hartung HP, Zettl UK (2016). Disease-modifying therapies and infectious risks in multiple sclerosis. Nat Rev Neurol.

[13] Klebe S, Stevanin G, Depienne C (2015). Clinical and genetic heterogeneity in hereditary spastic paraplegias: from SPG1 to SPG72 and still counting. Rev Neurol.

[14] Merrill JE, Hanak S, Pu SF, Liang J, Dang C, Iglesias-Bregna D, Harvey B, Zhu B, McMonagle-Strucko K (2009). Teriflunomide reduces behavioral, electrophysiological, and histopathological deficits in the Dark Agouti rat model of experimental autoimmune encephalomyelitis. J Neurol.

[15] Ringheim GE, Lee L, Laws-Ricker L, Delohery T, Liu L, Zhang D, Colletti N, Soos TJ, Schroeder K, Fanelli B (2013). Teriflunomide attenuates immunopathological changes in the dark agouti rat model of experimental autoimmune encephalomyelitis. Front Neurol.

[16] Nair AB, Jacob S (2016). A simple practice guide for dose conversion between animals and human. J Basic Clin Pharm.

[17] Lin B, Peng EB (2013). Retinal ganglion cells are resistant to photoreceptor loss in retinal degeneration. PLoS One.

[18] Sanes JR, Masland RH (2015). The types of retinal ganglion cells: current status and implications for neuronal classification. Annu Rev Neurosci.

[19] Kroner A, Ip CW, Thalhammer J, Nave KA, Martini R (2010). Ectopic T-cell specificity and absence of perforin and granzyme B alleviate neural damage in oligodendrocyte mutant mice. Am J Pathol.

[20] Groh J, Berve K, Martini R (2017). Fingolimod and teriflunomide attenuate neurodegeneration in mouse models of neuronal ceroid lipofuscinosis. Mol Ther.

[21] Groh J, Stadler D, Buttmann M, Martini R (2014). Non-invasive assessment of retinal alterations in mouse models of infantile and juvenile neuronal ceroid lipofuscinosis by spectral domain optical coherence tomography. Acta Neuropathol Commun.

[22] Faul F, Erdfelder E, Lang AG, Buchner A (2007). G*Power 3: a flexible statistical power analysis program for the social, behavioral, and biomedical sciences. Behav Res Methods.

[23] Dendrou CA, Fugger L (2017). Immunomodulation in multiple sclerosis: promises and pitfalls. Curr Opin Immunol.

[24] Groh J, Ribechini E, Stadler D, Schilling T, Lutz MB, Martini R (2016). Sialoadhesin promotes neuroinflammation-related disease progression in two mouse models of CLN disease. Glia.

[25] Dai H, Wan N, Zhang S, Moore Y, Wan F, Dai Z (2010). Cutting edge: programmed death-1 defines CD8+CD122+ T cells as regulatory versus memory T cells. J Immunol.

[26] Akane K, Kojima S, Mak TW, Shiku H, Suzuki H (2016). CD8+CD122+CD49dlow regulatory T cells maintain T-cell homeostasis by killing activated T cells via Fas/FasL-mediated cytotoxicity. Proc Natl Acad Sci U S A.

[27] Melzer N, Meuth SG (2014). Disease-modifying therapy in multiple sclerosis and chronic inflammatory demyelinating polyradiculoneuropathy: common and divergent current and future strategies. Clin Exp Immunol.

[28] Bar-Or A, Pachner A, Menguy-Vacheron F, Kaplan J, Wiendl H (2014). Teriflunomide and its mechanism of action in multiple sclerosis. Drugs.

[29] Dimitrova P, Skapenko A, Herrmann ML, Schleyerbach R, Kalden JR, Schulze-Koops H (2002). Restriction of de novo pyrimidine biosynthesis inhibits Th1 cell activation and promotes Th2 cell differentiation. J Immunol.

[30] Zeyda M, Poglitsch M, Geyeregger R, Smolen JS, Zlabinger GJ, Horl WH, Waldhausl W, Stulnig TM, Saemann MD (2005). Disruption of the interaction of T cells with antigen-presenting cells by the active leflunomide metabolite teriflunomide: involvement of impaired integrin activation and immunologic synapse formation. Arthritis Rheum.

[31] Miller AE (2017). Teriflunomide in multiple sclerosis: an update. Neurodegener Dis Manag.

[32] Metzler B, Gfeller P, Guinet E (2016). Restricting glutamine or glutamine-dependent purine and pyrimidine syntheses promotes human T cells with high FOXP3 expression and regulatory properties. J Immunol.

[33] Wiendl H, Gross C, Lindner M, Eschborn M, Weisser L, Posevitz-Fejfar A, Schulte-Mecklenbeck A, Van Wijmeersch B, Hupperts R, Brette S (2016). TERI-DYNAMIC: exploring the impact of teriflunomide on immune cell population size, receptor repertoire, and function in patients with RRMS (P5.282). Neurology.

[34] Koch MW, Cutter G, Stys PK, Yong VW, Metz LM (2013). Treatment trials in progressive MS—current challenges and future directions. Nat Rev Neurol.

[35] Lassmann H, van Horssen J, Mahad D (2012). Progressive multiple sclerosis: pathology and pathogenesis. Nat Rev Neurol.

[36] Nelson F, Lebrun-Frenay C, Camu W, Boyko A, Thangavelu K, Rufi P, Cavalier S, Truffinet P, Liang J, Lublin F (2016). Outcomes in patients with progressive MS: analysis of teriflunomide long-term extension data (P3.038). Neurology.

[37] Lublin F. Long-term disability outcomes in teriflunomide-treated patients in TEMSO and TOWER: an EDSS and FSS categorical analysis. 2017. http://onlinelibrary.ectrims-congress.eu/ectrims/2017/ACTRIMS-ECTRIMS2017/199735/fred.lublin.long-term.disability.outcomes.in.teriflunomide-treated.patients.in.html.

[38] Moser HW, Lazzarini RA, Griffin JW, Lassmann H, Nave KA, Miller RH, Trapp BD (2004). Adrenoleukodystrophies. Myelin biology and disorders.

[39] Barrette B, Nave KA, Edgar JM (2013). Molecular triggers of neuroinflammation in mouse models of demyelinating diseases. Biol Chem.

[40] Klebe S, Depienne C, Gerber S, Challe G, Anheim M, Charles P, Fedirko E, Lejeune E, Cottineau J, Brusco A (2012). Spastic paraplegia gene 7 in patients with spasticity and/or optic neuropathy. Brain.

