# Supplementary material for: Teriflunomide attenuates neuroinflammation-related neural damage in mice carrying human PLP1 mutations
Source: J Neuroinflammation. 2018 Jul 3;15:194. doi: 10.1186/s12974-018-1228-z (PMC6031103; doi:10.1186/s12974-018-1228-z)
Supplement: Supplementary file 1 — Figure S1. Lack of impact of teriflunomide treatment on blood leukocyte numbers. Figure S2. Confirmation of PD-1 expression as a marker of CD8+ CD122+ regulatory T cells in the CNS of PLPmut mice. Figure S3. Preventive but neither terminated nor therapeutic treatment impairs the increase of CD4+ T-lymphocyte numbers in PLPmut mice. Figure S4. Lack of evidence for an immune-unrelated effect of therapeutic teriflunomide treatment. Figure S5. Lack of evidence for an immune-unrelated effect of therapeutic teriflunomide treatment on histopathological features. Figure S6. Long-term preventive treatment does not reduce T-lymphocyte and microglia/macrophage numbers but leads to a reduction of activated Sn+ microglial cells. (PDF 1034 kb) [file 12974_2018_1228_MOESM1_ESM.pdf]

Additional file 1

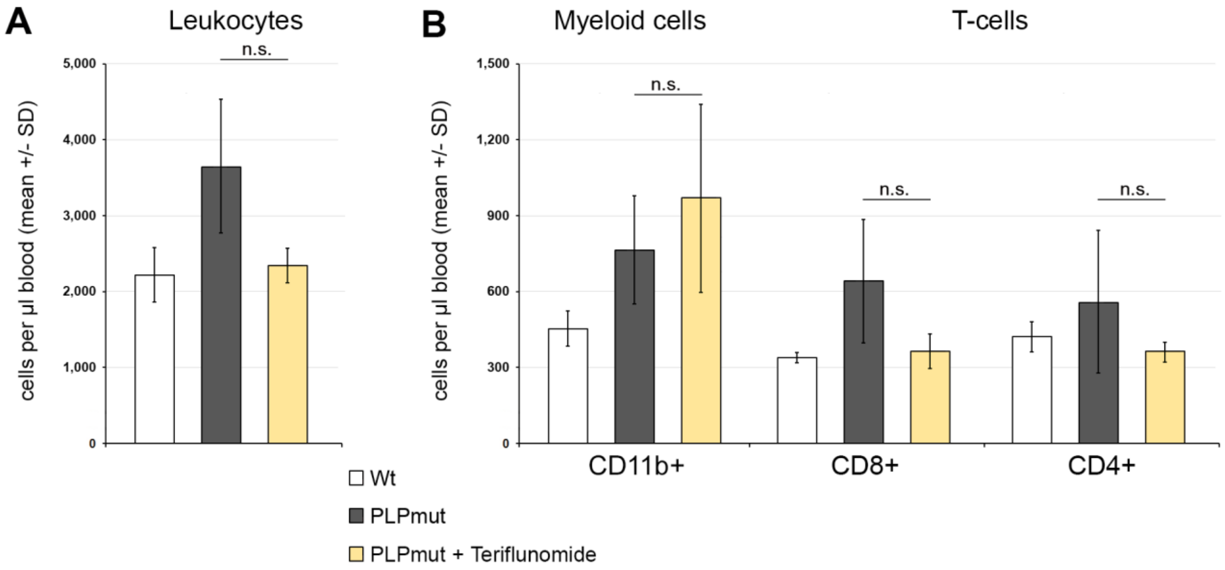

Figure S1

Lack of impact of teriflunomide treatment on blood leukocyte numbers.

(**A, B**) Effect of preventive treatment on total leukocyte (**A**) and on myeloid cell and T-lymphocyte numbers (**B**) in the peripheral blood. Based on flow cytometry, no significant changes of leukocyte numbers were detectable. One-way ANOVA and Tukey's *post hoc* tests. $n = 5$ mice per group.

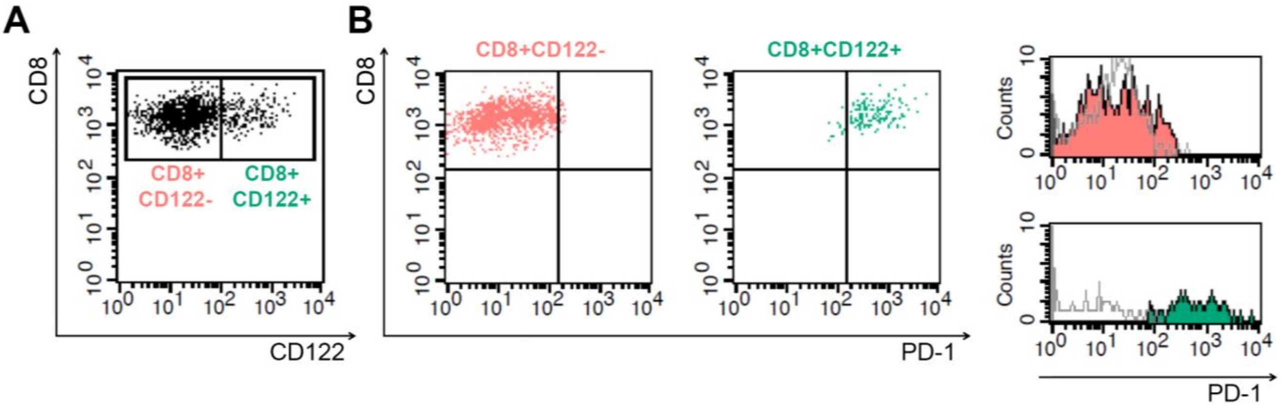

Figure S2

Confirmation of PD-1 expression as a marker of CD8+CD122+ regulatory T-cells in the CNS of *PLPmut* mice.

(**A**) Representative example of flow cytometric analysis of T-lymphocytes freshly isolated from brains of untreated *PLPmut* mice gated for CD8 and CD122 expression. The majority of the CNS-derived CD8+ T-cells are CD122- effector cells (red); only few cells are double-positive for CD8 and CD122 (green) and thus regulatory T-cells. (**B**) Representative example of flow cytometric analysis of gated CD8+CD122- (red) and CD8+CD122+ (green) T-lymphocytes from brains of untreated *PLPmut* mice analysed for PD-1-coexpression. Only CD8+CD122+ regulatory cells coexpress PD-1. Right histograms display corresponding counts for CD8+CD122- (red) and CD8+CD122+ cells (green) and their coexpression of PD-1. Appropriate isotype control counts are represented in grey.

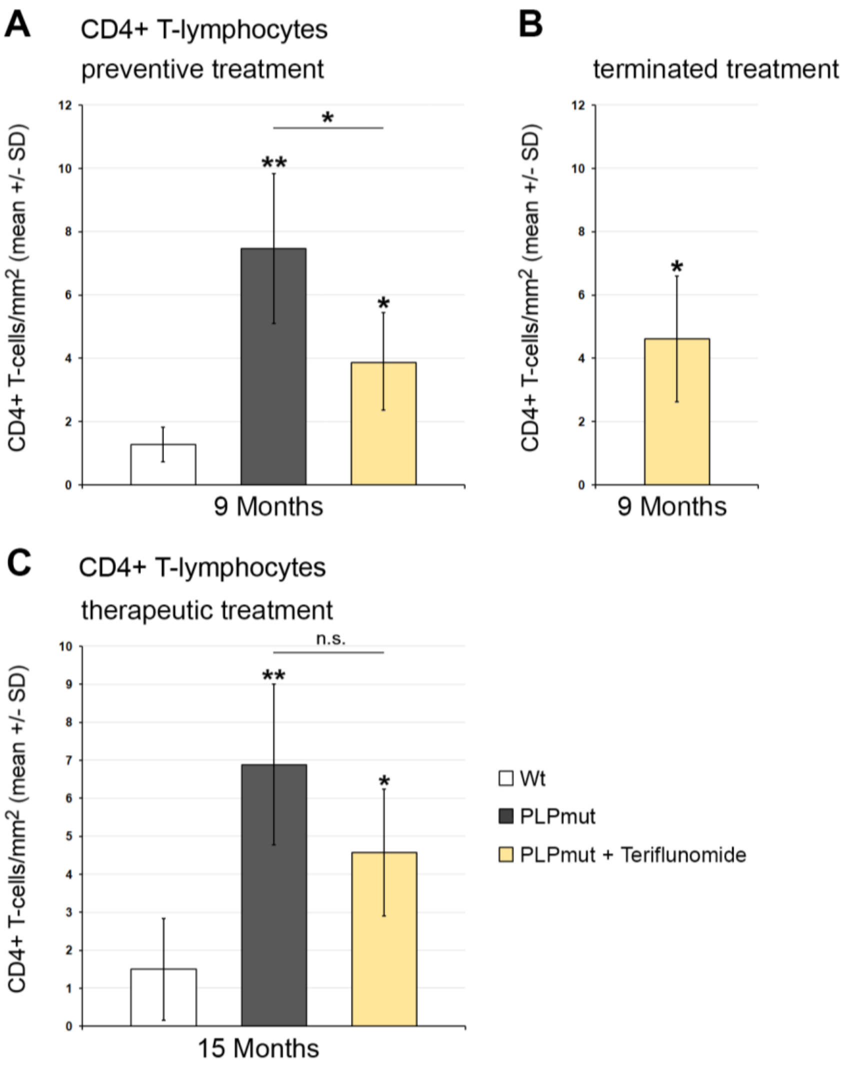

Figure S3

Preventive, but neither terminated nor therapeutic treatment impairs the increase of CD4+ T-lymphocyte numbers in *PLPmut* mice.

(**A**) Quantification of CD4+ T-lymphocytes in optic nerve sections of *Wt* and *PLPmut* mice and in *PLPmut* mice after 150 days of preventive treatment. Like CD8+ T-lymphocytes (Figure 2), the numbers of CD4+ T-cells were significantly increased in the untreated *PLPmut* mice; this elevation was attenuated by preventive treatment. (**B**) Quantification of CD4+ T-lymphocytes in optic nerve sections of *PLPmut* mice after terminated treatment. Treatment termination did not lead to an overshoot or rebound of T-cell numbers, but failed to significantly preserve reduction of T-cell numbers. (**C**) Quantification of CD4+ T-lymphocytes in optic nerve sections of *Wt* and *PLPmut* mice and in *PLPmut* mice after 150 days of therapeutic treatment. Therapeutic treatment did not significantly reduce the increase of CD4+ T-cell numbers in *PLPmut* mice. Note generally lower numbers of CD4+ T-

cells in comparison to CD8+ T-cells (compare to Figure 2). One-way ANOVA and Tukey's *post hoc* tests. *$P < 0.05$, **$P < 0.01$. $n = 5$ mice per group.

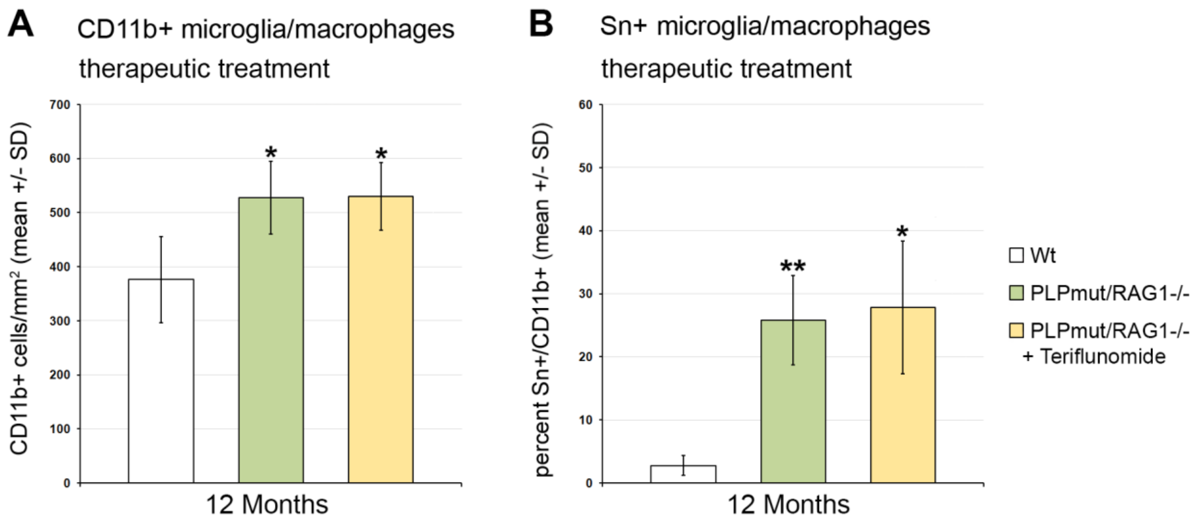

Figure S4

Lack of evidence for an immune-unrelated effect of therapeutic teriflunomide treatment.

(**A**) Quantification of CD11b+ and (**B**) Sn+ activated microglia/macrophages in optic nerves of *Wt*, *PLPmut*/*Rag1*$^{-/-}$ and therapeutically-treated *PLPmut*/*Rag1*$^{-/-}$ mice. Treatment had no further effect on (in comparison with *PLPmut* reduced) CD11b+ or Sn+ microglia/macrophage numbers in *PLPmut* mice lacking mature adaptive immune cells. One-way ANOVA and Tukey's *post hoc* tests. *$P < 0.05$, **$P < 0.01$. $n = 5$ mice per group.

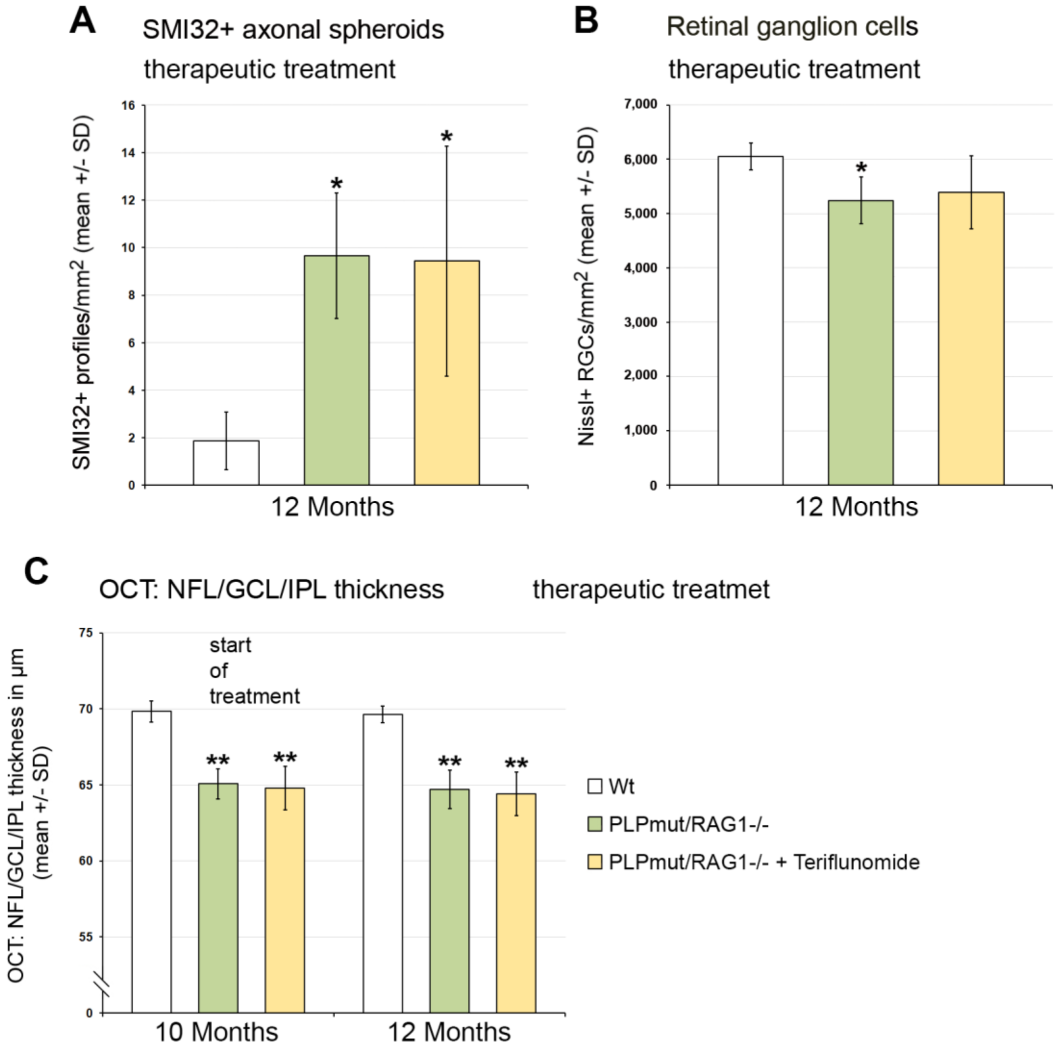

Figure S5

Lack of evidence for an immune-unrelated effect of therapeutic teriflunomide treatment on histopathological features.

(**A**) Quantification of SMI32+ axonal spheroids in optic nerves, (**B**) of cresyl violet-stained retinal ganglion cells and of inner retinal composite layer thickness in *Wt*, *PLPmut*/*Rag1*$^{-/-}$ and therapeutically-treated *PLPmut*/*Rag1*$^{-/-}$ mice by OCT. Treatment failed to further ameliorate the corresponding histopathological alterations which have been attenuated by RAG1-deficiency. One-way ANOVA and Tukey's *post hoc* tests. **P* < 0.05, ***P* < 0.01. *n* = 5 mice per group.

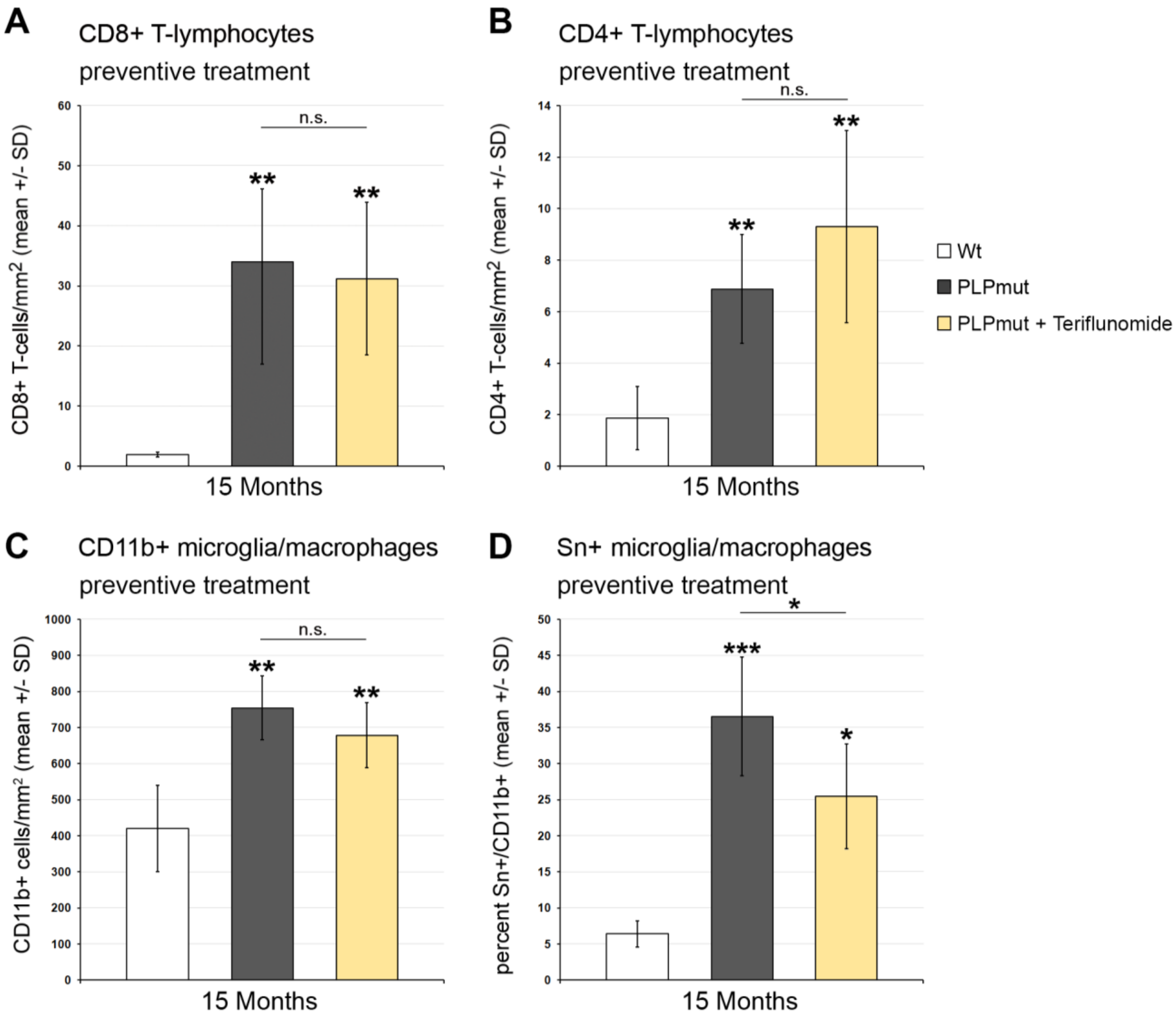

Figure S6

Long-term preventive treatment does not reduce T-lymphocyte and microglia/macrophage numbers but leads to a reduction of activated Sn+ microglial cell numbers.

(**A**) Quantification of CD8+ T-lymphocytes, (**B**) CD4+ T-lymphocytes, (**C**) total numbers of microglia/macrophages and (**D**) Sn+ microglial cells in longitudinal sections of optic nerves of *Wt*, *PLPmut* and *PLPmut* mice after long-term preventive treatment. Only activated Sn+ microglial cell numbers were still reduced after long-term treatment conditions. One-way ANOVA and Tukey's *post hoc* tests. $^{*}P < 0.05$, $^{**}P < 0.01$, $^{***}P < 0.001$. $n = 5$ mice per group.
